# Open access resources for genome-wide association mapping in rice

**DOI:** 10.1038/ncomms10532

**Published:** 2016-02-04

**Authors:** Susan R. McCouch, Mark H. Wright, Chih-Wei Tung, Lyza G. Maron, Kenneth L. McNally, Melissa Fitzgerald, Namrata Singh, Genevieve DeClerck, Francisco Agosto-Perez, Pavel Korniliev, Anthony J. Greenberg, Ma. Elizabeth B. Naredo, Sheila Mae Q. Mercado, Sandra E. Harrington, Yuxin Shi, Darcy A. Branchini, Paula R. Kuser-Falcão, Hei Leung, Kowaru Ebana, Masahiro Yano, Georgia Eizenga, Anna McClung, Jason Mezey

**Affiliations:** 1 School of Integrative Plant Sciences, Plant Breeding and Genetics section, Cornell University, Ithaca, 14850 New York, USA; 2 Department of Biological Statistics and Computational Biology, Cornell University, Ithaca, 14850 New York, USA; 3 International Rice Research Institute, DAPO Box 7777, 1301 Metro Manila, Philippines; 4 School of Food Science, University of Queensland, St Lucia, 4072 Queensland, Australia; 5 Transnational Learning Center, Cornell University, Ithaca, 14850 New York, USA; 6 National Institute of Agrobiological Sciences, 2-1-2 Kannondai, Tsukuba, 305-8602 Ibaraki, Japan; 7 USDA–ARS Dale Bumpers National Rice Research Center, 2890 Hwy. 130 E., Stuttgart, Arkansas 72160, USA

## Abstract

Increasing food production is essential to meet the demands of a growing human population, with its rising income levels and nutritional expectations. To address the demand, plant breeders seek new sources of genetic variation to enhance the productivity, sustainability and resilience of crop varieties. Here we launch a high-resolution, open-access research platform to facilitate genome-wide association mapping in rice, a staple food crop. The platform provides an immortal collection of diverse germplasm, a high-density single-nucleotide polymorphism data set tailored for gene discovery, well-documented analytical strategies, and a suite of bioinformatics resources to facilitate biological interpretation. Using grain length, we demonstrate the power and resolution of our new high-density rice array, the accompanying genotypic data set, and an expanded diversity panel for detecting major and minor effect QTLs and subpopulation-specific alleles, with immediate implications for rice improvement.

## Abstract (web summary)

Understanding the link between genotype and phenotype can facilitate efforts by breeders to utilize natural variation and develop new crop varieties. Here the authors present a diverse germplasm collection, a high-density genotyping array and a set of bioinformatic tools to enable association mapping in rice.

## Graphical abstract

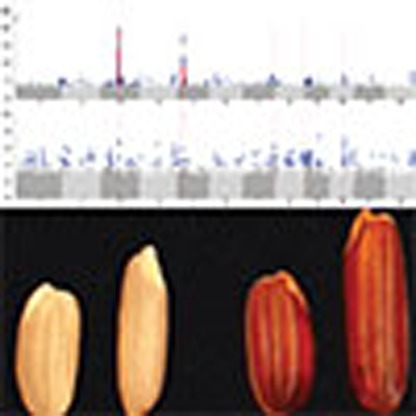

Rice (*Oryza sativa*) is a staple food that feeds 3 billion people^1^,^2^. It has the largest, publicly available, single-species germplasm collection in the world^3^, and a rich ‘omics' toolkit. Rice was the first crop plant to be fully sequenced^4^, has over 3,000 re-sequenced varieties^5^,^6^,^7^,^8^, *de novo* assemblies within three subpopulations^9^,^10^,^11^ and BAC-based sequence assemblies for 12 related *Oryza* species^12^. With these resources in hand, the challenge is to utilize this information to understand and predict how genotypic variation gives rise to the abundance of phenotypic variation that we see in nature and in agriculture.

Genome-wide association studies (GWAS) offer a powerful strategy for understanding the genetic basis of complex traits. This approach has been particularly productive for rice, where major associations have been detected for important traits, including grain size, grain weight, flowering time, disease resistance and abiotic stress tolerance, among others^13^,^14^,^15^,^16^. Hundreds of genes governing traits of interest have been cloned and new, powerful tools are currently being deployed to achieve targeted genome editing^17^,^18^,^19^. In applied breeding programs, major-effect loci discovered via quantitative trait loci (QTL) mapping and GWAS have been successfully introgressed from wild and exotic genetic resources into elite, high-yielding varieties via marker-assisted selection^20^,^21^,^22^.

Rice has been particularly amenable to GWAS largely because of its evolutionary and domestication history. The wild ancestor of Asian rice, *O. rufipogon*, is a partially (35%) outcrossing species complex broadly dispersed across tropical South, South-East and Eastern Asia. It consists of locally or regionally adapted subpopulations with both perennial and annual forms^23^. The domestication of *O. sativa* began ∼10,000–12,000 years ago, and human selection was accelerated by inbreeding which retained and fixed many favourable alleles of large effect. The unique combinations of locally adapted allele complexes in different subpopulations provide ideal characteristics for GWAS, including both universal and population-specific large effect alleles, extended linkage disequilibrium (LD) within some subpopulations that is advantageous for mapping with low marker coverage, rapid LD decay within other subpopulations offering improved mapping resolution and an abundance of well-partitioned genetic variation among subpopulations. These properties, in combination with a small, tractable genome (380 Mb), and the ability to produce immortal panels comprised of thousands of inbred, homozygous individuals, make it possible to evaluate the same traits in different environments simultaneously, and to evaluate a variety of different traits on the same germplasm over time. The ability to make controlled matings and to generate large populations each generation provides a powerful system for validating loci and for harnessing their value by introgressing favourable alleles into other genetic backgrounds.

Here, we report the development of an association mapping panel consisting of 1,568 diverse inbred rice varieties and a genotyping data set generated using a high-density rice array (HDRA) comprised of 700,000 single-nucleotide polymorphisms (SNPs). The HDRA represents the most densely populated genotyping array available for any organism at this time, based on the number of SNPs/kb interrogated across the genome. We explore the value of these resources for GWAS using grain length as the phenotype, and use numerous analytical techniques to identify significant associations that uncover both major and minor QTLs. Further, we demonstrate distinct advantages of cross-population mapping where we gain power by directly accounting for large effect alleles from within the mapping model. We show how this may lead to the identification of population-specific haplotypes, which are of particular value to the plant breeder.

The GWAS resources and data sets generated in this project are designed to facilitate the collection of phenotypic information on a wide range of traits and characteristics (see companion paper by Crowell *et al.*^24^) and to rapidly and efficiently convert that information into focused hypotheses about causal genes and QTL locations. They also provide a bridge between genomics and applied plant breeding. With increasing accuracy over time, data sets generated for different purposes and at differing levels of resolution can be leveraged against each other via shared haplotypes, making it feasible to undertake cross-population imputation of both genotype and phenotype and to predict the phenotypic outcomes from genotypic data. GWAS results can also be incorporated as fixed variables into genomic selection models to improve the accuracy of genomic estimated breeding values^25^,^26^,^27^.

These resources are part of a larger rice diversity research platform being developed by the international rice research community. All the materials, data sets and tools reported here are available as open-source products. Together these resources provide the basis for further exploration of phenotype–genotype relationships in rice and for expediting variety development in this important food crop.

## Results

### The high-density rice array

The HDRA assays 700,000 SNPs (average call rate=93.5%), or ∼1 SNP every 0.54 kb across the rice genome. It was designed to efficiently capture most of the haplotype variation observed in a discovery panel consisting of 16M SNPs (generated by sequencing 125 rice genomes at ∼7 × genome coverage) and to maximize the inclusion of non-synonymous SNPs (see Supplementary Note 1). An estimated 45% of HDRA SNPs map within genes, covering all 39,045 unique, non-TE rice gene models (http://rice.plantbiology.msu.edu/), while 55% of SNPs map to intergenic regions (Supplementary Fig. 1). Non-synonymous SNPs, which represent ∼16% of all informative SNPs on the HDRA (Table 1), are found in 91% of unique, non-TE gene models. Of the genic SNPs, 55% are distributed within exons, 36% within introns, 3% within 5′-untranslated regions (UTRs) and 6% within 3′-UTRs. Of the intergenic SNPs, 50% are located in putative regulatory regions (PPRs) within 2 kb of a transcriptional start site.

### Genetic structure of the rice diversity panel

Using the HDRA, we assayed SNP variation in a collection of 1,568 diverse accessions of *O. sativa* (Supplementary Data 1) and the resulting genotypic data set was analyzed for population structure using fastSTRUCTURE^28^. The optimum number of subpopulations was determined to be K=5. The five subpopulations were identified as *aus*, *indica*, *tropical japonica*, *temperate japonica* and *aromatic* (or *Kashmiri japonica*, Group V), with *aus* and *indica* comprising the *INDICA* varietal group, and *tropical japonica*, *temperate japonica* and *aromatic* comprising the *JAPONICA* varietal group^15^,^16^,^29^,^30^. (We use upper-case letters to indicate the two major varietal groups, *INDICA* and *JAPONICA*, and lower-case letters to indicate subpopulations.) Using a cutoff of 70% ancestry, accessions were classified into one of the five major subpopulation groups, or into one of three admixture classes: admixed *INDICA* (*aus-indica*), admixed *JAPONICA* (*tropical japonica-temperate japonica-aromatic*) or admixed *INDICA-JAPONICA* (Supplementary Data 1).

**Figure 1:**
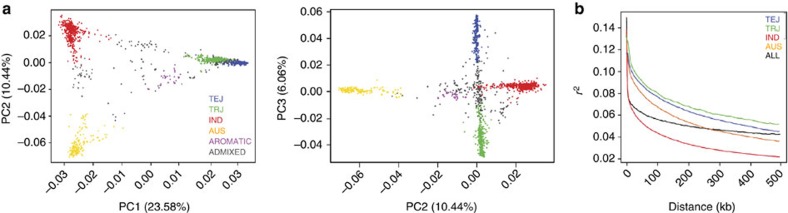
Genetic structure of the rice diversity panel. (**a**) PPCA showing global genetic variation in the rice diversity panel. Left: first and second PCs; right: second and third PCs. (**b**) LD as a function of distance between SNP markers in each *O. sativa* subpopulation. The circles in **a** and lines in **b** are coloured based on subpopulation assignments from FastSTRUCTURE: IND—*indica* (red); AUS—*aus* (yellow); TEJ—*temperate japonica* (blue); TRJ—*tropical japonica* (green); ALL—all subpopulations combined (black). Purple circles: aromatic; Grey circles: admixed lines.

We applied principal component analysis^31^ to summarize global genetic variation in the diversity panel (Fig. 1a). The top three principal components (PCs) explain 40% of the genetic variation, where the first PC separates the *INDICA* and *JAPONICA* varietal groups (23.58% of the variation), the second PC separates the *aus* and the *indica* subpopulations (10.44%), while the third PC separates the three *JAPONICA* subpopulations (6.06%). LD decays rapidly with distance in all populations (Fig. 1b), with *indica* exhibiting the most rapid LD decay and *temperate japonica* the most extended LD.

The design of the HDRA provides a rich selection of shared and private SNPs distributed across the subpopulations of *O. sativa* and its closest wild relatives, *O. rufipogon/O. nivara*, with emphasis on efficiency of haplotype detection. A total of 671,906 SNPs detected polymorphism within the subpopulations of *O. sativa*, ∼8% were private to individual subpopulations (Table 1), and 23,748 SNPs were private to the Asian wild progenitors. When subpopulation statistics were compared, the largest number of both private and shared SNPs was detected within *indica*, the smallest was detected in *aromatic* and the other subpopulations had intermediate numbers of informative SNPs on the array (Table 1; Supplementary Data 2). These figures are consistent with sequence-based estimates of relative subpopulation diversity^5^,^15^.

There is a non-linear, decreasing relationship between the number of lines assayed and SNPs detected, indicating that the same SNPs are being assayed over and over again as the panel size increases. The diversity panel developed by the NIAS^32^, by far the smallest collection of lines, is very efficient for its size in capturing a broad range of diversity, while the size of the two larger rice diversity panels, RDP1 (refs 16, 33) and RDP2 (first introduced here; Supplementary Data 1) enhance the resolution of GWAS primarily by assaying new recombinants.

**Figure 2:**
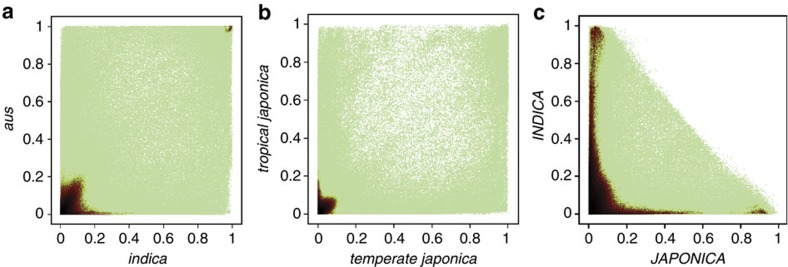
Allele frequencies in the different subpopulations of *O. sativa*. Two-dimensional site frequency spectra (SFS) for the entire diversity panel (RDP1 and 2): (**a**) *indica* vs *aus*; (**b**) *temperate japonica* vs *tropical japonica*; (**c**) *JAPONICA* vs *INDICA*. The spectra were displayed by grouping frequency values for each SNP in a pair of populations into 250 bins using the hexbin package in R. Note: the joint frequency of the *indica* and *japonica* samples cannot exceed 0.5 because without an outgroup, we calculate the frequency of the minor allele in the entire RDP1 and 2 sample (that is, the frequency spectrum is folded). However, in different sub-samples (for example, within *japonica*) some alleles with frequencies below 0.5 in the complete sample can be more prevalent, even reaching fixation (frequency of 1.0), thus giving the appearance of an unfolded SFS.

Given that diversity is partitioned into deeply differentiated subpopulations in *O. sativa*, we further explored the genetic relationships between pairs of subpopulations by examining 2D site frequency spectra. The majority of SNPs that are at low frequency in *indica* are also found at low frequency in *aus*, though *indica-*specific and *aus-*specific SNPs are also detected (Fig. 2a). Similarly, SNPs that are at low frequency in *tropical japonica* are also usually observed at low frequency in *temperate japonica*, with few exceptions (Fig. 2b). However, we see a very different picture when we compare the *INDICA* and *JAPONICA* varietal groups, where the sites that are at moderate-to-high frequency in one of the subpopulations are found at markedly lower frequencies in the other (Fig. 2c). Nonetheless, a common set of 291,132 SNPs (43%) segregate cross all 5 subpopulations of *O. sativa*, while 418,502 SNPs segregate in the *INDICA* varietal group and 295,984 SNPs segregate in the *JAPONICA* varietal group. This made it possible to perform GWAS using different subsets of germplasm, though many SNP frequencies vary significantly across the different populations. Interestingly, the frequency distribution of synonymous and non-synonymous SNPs is very similar across groups, with slightly more non-synonymous SNPs occurring at low frequency, but a good distribution of both across the entire site frequency spectra (Supplementary Fig. 2). The observed SNP frequencies and distributions within and between subpopulations have important implications for the way GWAS is conducted in rice.

### Improved GWAS resolution using a larger number of lines

In this study we investigated the potential to resolve both major- and minor-effect GWA-QTLs using the new HDRA genotypic data set and grain length as a phenotype. Grain length is a highly heritable phenotype, with broad sense heritability of 0.976. A major QTL for grain size was previously identified on rice chromosome 3 in a number of studies across different genetic backgrounds and environments^34^,^35^. The gene underlying this QTL, *GRAIN SIZE 3* (*GS3*, Os03g0407400), has been cloned and characterized and the most common functional polymorphism is a C≥A transition that occurs in the second exon of the gene. Using genetic transformation, Takano-Kai *et al.*^36^ demonstrated that the wild-type allele (C) confers short grain and the derived allele (A) confers long grain in natural populations. Other mutations in the same gene have also been shown to affect grain length but they occur at low frequency in *O. sativa*, making them unlikely to be detected using GWAS^37^.

Rice chromosome 5 carries two closely linked genetic loci associated with grain size that have been positionally cloned and functionally characterized, namely *GRAIN WIDTH 5* (*GW5*) and *GRAIN SIZE 5* (*GS5*, LOC_Os05g06660)^38^,^39^. For *GW5*, the functional polymorphism is a 1,212 bp deletion in the reference genome (cv. Nipponbare) that removes the only exon of the gene, resulting in a short, wide, heavy grain^38^,^40^. For *GS5*, polymorphisms in the promoter region leading to differential gene expression were associated with grain size^39^. In addition, a vast number of QTL regions have been mapped, and 14 genes associated with grain shape and size have been cloned^41^,^42^,^43^,^44^,^45^,^46^,^47^,^48^,^49^,^50^,^51^,^52^,^53^,^54^,^55^ (Supplementary Data 4). Although the function of many of these genes is still poorly understood, they provide us with clear, true-positive loci where common alleles affecting grain size can serve as the basis for assessing the mapping power and resolution of the HDRA.

**Figure 3:**
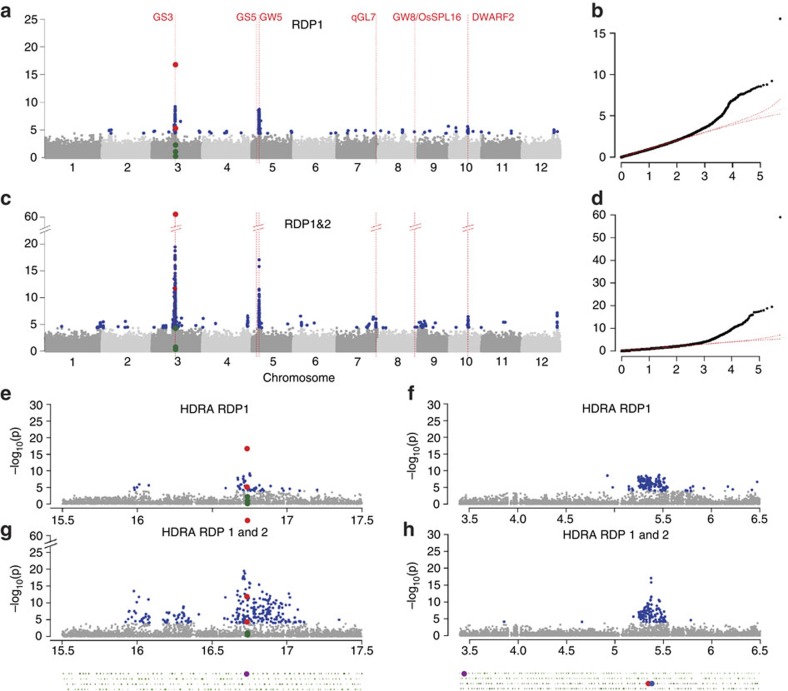
GWAS for rice grain length. Manhattan plots (**a**,**c**) and quantile–quantile plots (**b**,**d**) for GWAS using the HDRA on RDP1 (**a**,**b**); and GWAS using the HDRA on RDP1 and 2 (**b**,**d**). On each Manhattan plot, the *x*-axis shows the SNPs along each chromosome; *y*-axis is the −log10 (*P* value) for the association. Red vertical lines indicate the position of candidate genes and/or QTL regions associated with grain length. (**e**,**g**) Zoom-in on the major GWAS peaks for grain length on chromosomes 3 (15.5–17.5 Mb) using (**e**) the HDRA on RDP1; and (**g**) the HDRA on RDP1 and 2. Zoom-in of the major GWAS peaks for grain length on chromosomes 5 using (**f**) the HDRA on RDP1; and (**h**) the HDRA on RDP1 and 2. Green circles underneath each set of plots represent gene models; the gene model highlighted in magenta in **e**,**g** is *GS3* (LOC_Os03g0407400); gene models highlighted in **f**,**h** are *GS5* (LOC_Os05g06660, magenta) and the two gene models flanking the *GW5* deletion, LOC_Os05g09510 (blue) and LOC_Os05g09520 (red). In all panels, significant SNPs (with *P* values at or above the 10% FDR threshold) are coloured blue. In **a**,**c**,**e**,**g**, the SNPs that fall within the coding region of *GS3* are highlighted in green if they have non-significant *P* values and in red if they have significant *P* values. The SNPs highlighted are (SNP name (*P* value for RDP1, *P* value for RDP1 and 2)): SNP-3.16728637 (0.69, 0.50); SNP-3.16730230 (1.00, 0.21); SNP-3.16732086 (1.83e−17, 1.05 e−59); SNP-3.16732418 (6.25 e−06, 1.96 e−12); SNP-3.16733169 (0.01, 7.02 e−05) and SNP-3.16733346 (0.12, 0.42).

Using the HDRA on the RDP1 panel alone, or the RDP1 and 2 panels together, we see two major GWAS peaks associated with grain length, one on chromosome 3 and one on chromosome 5 (Fig. 3a). Zoom-in plots of the peaks on chromosome 3 confirm that the same peaks are detected, and SNP-3.16732086 is the most significant SNP (MS-SNP) in both cases (Fig. 3b). With RDP1 alone, the MS-SNP explains 16.5% of the phenotypic variation and is detected at *P* value=4.48e−17, while when RDP1 and 2 are used, the MS-SNP is detected at *P* value=1.48e−55 and explains 15.9% of the variation (Fig. 3b). The highly significant *P* values are because of the fact that SNP-3.16732086 directly interrogates the functional polymorphism in exon 2 of *GS3*, while the second and third MS-SNPs map 11.3 kb (SNP 3.16718013) and 16.8 kb (SNP 3.16712500) upstream of the transcriptional start site of *GS3*, and explain 6% and 5.8% of the phenotypic variation, respectively (Supplementary Data 3). Interestingly, in addition to SNP-3.16732086, there are several HDRA SNPs that map within the *GS3* gene itself (coloured green in Fig. 3a,b) but have significance levels and allele effects similar to SNPs that are farther away. This is because of the fact that, despite being within the causal gene, they are not in perfect LD with the functional SNP that is responsible for grain length.

Turning our attention to the peak on chromosome 5, a cursory comparison of the two plots suggests that they detect a GWA peak in roughly the same region (Fig. 3c). Using the HDRA on RDP1, a cluster of significant SNPs forms a dense plateau across a ∼300 kb region, while the resolution is greatly improved with the larger panel of germplasm (RDP1 and 2).

**Figure 4:**
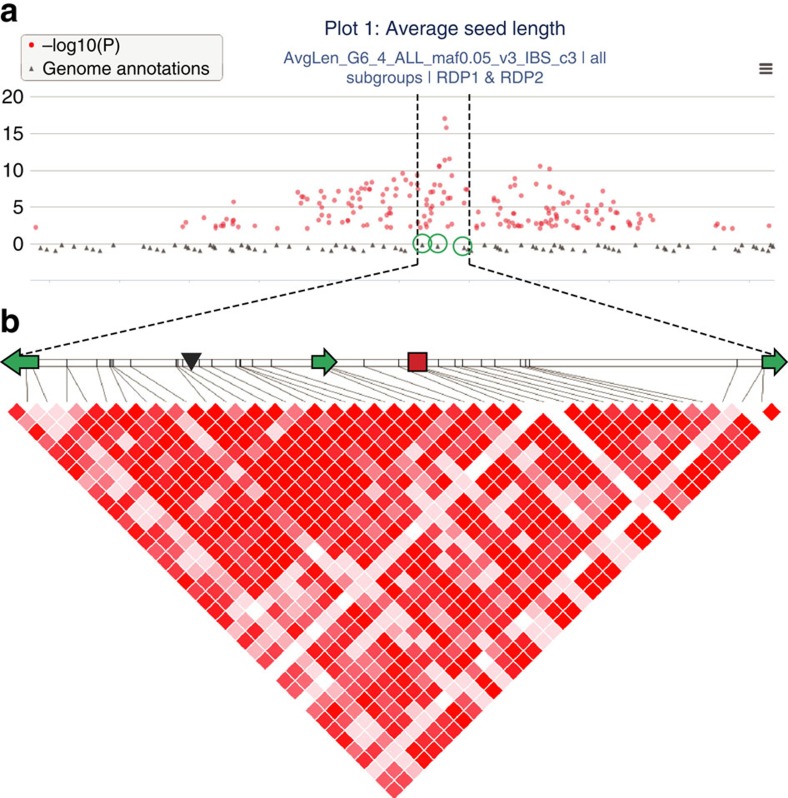
GWAS peak on chromosome 5 in relation to the *GW5* functional deletion. (**a**) screenshot from our GWAS Viewer (ricediversity.org) zoomed-in to show GWAS peak over *GW5* candidate gene region in RDP1 and 2 (*ALL*). Three gene models under the peak are circled in green. (**b**) LD in the region of the *GW5* functional polymorphism. The horizontal bar shows a physical map of chromosome 5 (5.35–5.39 Mb); green arrows represent the three genes circled above, from left to right: LOC_Os05g09510, LOC_Os05g09520 and LOC_Os05g09530, with direction indicating strand orientation; position of the *GW5* functional deletion is shown as black triangle, and red square represents position of the MS-SNP (SNP-5.5371749, located at 5,371,772).

The grain length genes *GS5* and *GW5* are located 1.9 Mb apart on rice chromosome 5. The known functional polymorphism associated with *GW5* corresponds to a 1.2 kb deletion in the reference genome that deletes the *GW5* gene^40^. The deletion is located between LOC_Os05g09510 and LOC_Os05g09520 (Fig. 4). When we verified the distance of the MS-SNPs to *GW5* and *GS5*, the MS-SNP maps only 11 kb away from the *GW5* deletion (Fig. 4) and all the MS-SNPs cluster in the vicinity of the functional deletion affecting *GW5*; no peak was observed at *GS5* (Fig. 3c). This is consistent with the fact that the functional polymorphisms at *GS5* are known to be rare in rice germplasm^39^ and provides evidence that GWAS can distinguish levels of statistical support for two *a priori* candidate genes that are only 2 Mb apart.

We previously reported a GWA study in the RDP1 using a custom-designed genotyping array with 44,100 SNPs (44 K array)^16^. Although the 44 K array also detected the same two major peaks on chromosomes 3 and 5 (Supplementary Fig. 3), significant SNPs are sparse and tend to have lower *P* values than those detected by the HDRA.

### GWAS models with SNP covariates

**Figure 5:**
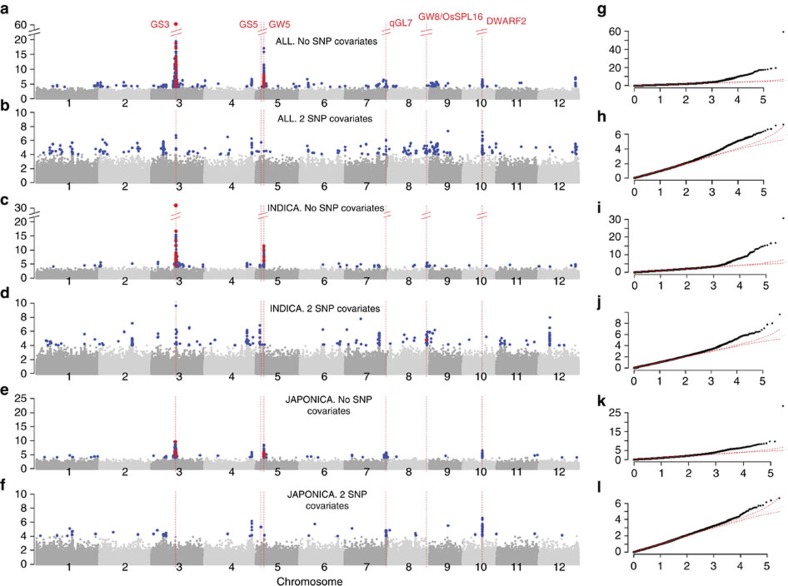
GWAS for grain length in the *Indica* and *Japonica* varietal groups and using SNP covariates. Manhattan plots (**a**–**f**) and quantile–quantile plots (**g**–**l**) for GWAS using the HDRA on RDP1 and 2. On each Manhattan plot, the *x*-axis shows the SNPs along each chromosome; *y*-axis is the −log10 (*P* value) for the association. Significant SNPs (with *P* values at or above the 10% FDR threshold) are coloured blue; significant SNPs within a 24 kb window surrounding candidate grain length genes are coloured red. Red vertical lines indicate the position of candidate genes and/or QTL regions associated with grain length. HDRA on RDP1 and 2 *ALL*, with 0 (**a**,**g**) and 2 SNP covariate (**b**,**h**): SNP-3.16732086 and SNP-5.5371749; HDRA on RDP1 and 2 *INDICA*, with 0 (**c**,**i**) and 2 SNP covariate (**d**,**j**): SNP-3.16732086 and SNP-5.5359497; HDRA on RDP1 and 2 *JAPONICA*, with 0 (**e**,**k**) and 2 SNP covariate (**f**,**l**): 3.16732086 and SNP-5.5366680.

To further explore the relationships among QTLs for grain length, we re-ran GWAS analyses for the HDRA on RDP1 and 2 and included the MS-SNPs (one from each major peak on chromosomes 3 and 5) as co-variates in the GWA models. When all samples were evaluated together, additional peaks were detected on all 12 rice chromosomes, with the most significant located on chromosome 10 (Fig. 5a). The MS-SNP defining the chromosome 10 peak also had the highest per cent phenotypic variation explained (PVE), but not the largest allele effect in *ALL* with two SNP covariates (Supplementary Data 3).

We also performed GWAS independently for the two varietal groups, *INDICA* (*aus+indica*+admixed *aus-indica*) and *JAPONICA* (*temperate japonica*, *tropical japonica*, *aromatic*+admixed *temperate-tropical-aromatic*; Fig. 5b,c), as well as for each subpopulation individually (Supplementary Fig. 4). From a bird's eye view, the same major GWAS peaks on chromosomes 3 and 5 were detected in the joint *INDICA* and joint *JAPONICA* analyses as described previously. Peaks were defined as regions containing at least one SNP at *P*<1.0e−05 and at least two additional SNPs (*P*<1.0e−4) located within a 100-kb window on either side of the MS-SNP. The functional SNP in the *GS3* gene (SNP 3.16732086) on chromosome 3 was consistently identified as the MS-SNP with the highest PVE and allele effect in all analyses, while on chromosome 5, different but closely linked SNPs (mapping within ∼12 kb of each other) identified the same peak in different analyses (Fig. 5, Supplementary Data 3). When the two MS-SNPs (from chromosomes 3 and 5) were used as covariates in each analysis, significant GWAS-peaks for grain length were detected in 12 regions of the genome in *ALL*, 13 regions in the joint *INDICA* analysis, and 2 regions in the joint *JAPONICA* analysis (Fig. 5, Supplementary Data 3). One of the 13 peaks identified in *INDICA* (on chromosome 8) and both of the peaks detected in *JAPONICA* were also identified in *ALL*, but no peaks were shared between *INDICA* and *JAPONICA*. This can be explained by the differences in allele frequencies, allele effects and per cent variance explained by the SNPs associated with grain length in each group.

An examination of the peaks in each of the individual subpopulations revealed novel features (Supplementary Fig. 4a–e). In the *indica* and *tropical japonica* subpopulations, the two major peaks on chromosomes 3 and 5 were clearly visible, while in *aus*, a minor peak was identified several kb away from *GS3* and no peak was observed near *GW5*. In *temperate japonica*, a very wide peak was observed 1.4 Mb to the left of *GS3* along with a minor peak at *GW5*. These results suggest the possibility that different causal genes in the *GS3* region may contribute to the observed phenotypic variation in the *aus* and *temperate japonica* subpopulations. When SNP covariates were applied to the subpopulation-specific analyses, we discovered additional minor peaks associated with grain length in each. Notably, *aus* had twice as many significant minor peaks as *indica* (along with higher average allele effect and PVE values and *indica* had twice as many as either *tropical* or *temperate japonica* (Supplementary Data 3). Half of the minor peaks in the subpopulation-specific analyses overlapped with those observed in *ALL* or in the joint-*INDICA* or joint-*JAPONICA* GWAS, but the other half were novel, providing interesting targets for further research and use in breeding. The GWAS results discussed here can be visualized at high resolution using our interactive browser and GWAS Viewer, as described in Supplementary Note 2.

In many cases, GWAS peaks were found to encompass previously described genes or QTLs associated with grain length or shape (Supplementary Data 4). A minor peak on chromosome 3 in the *indica* subpopulation co-localizes with *POSITIVE REGULATOR OF GRAIN LENGTH* 1 (*PGL1*); *PGL1* encodes an atypical non-DNA-binding bHLH that interacts *in vivo* with *ANTAGONIST OF PGL1* (*APG*) and increases grain length and weight in transgenic rice. *PGL1-APG* potentially represents a new grain length- and weight-controlling pathway^56^,^57^. A minor peak on chromosome 2 in *aus* co-localizes with *qLWR2*, a QTL for length-to-width ratio detected in a cross between an *indica* and a *japonica*^43^. A peak on chromosome 7 detected both in *ALL* and in *tropical japonica* co-localizes with a fine-mapped QTL (*qGL7*) controlling grain length, also detected in an *indica* X *japonica* cross^43^. A peak on chromosome 5 in *aus* is located in-between the candidate genes *APG*^57^ and *SGL/SRS3*^44^,^53^. *Short Grain Length* (*SGL*) is the causal gene of a small and round seed mutant (*sgl*/*srs3*, obtained from a tissue culture explant of *japonica* variety, Kitaake) and encodes a protein member of the kinesin 13 family. On chromosome 8, a peak detected in *ALL* as well as in the *aus* subpopulation co-localizes with the grain size gene *GW8*/*OsSPL16* (ref. 42). This gene encodes a SQUAMOSA promoter-binding protein. The allele conferring long grain at this locus was discovered in a *basmati* variety, suggesting that it may be shared between *aromatic* and *aus* varieties^34^,^41^,^58^. Finally, a broad peak was detected on chromosome 10 in *ALL*, encompassing the *DWARF2* gene ∼240 kb from the MS-SNP. This gene encodes a cytochrome P450 oxidoreductase involved in brassinosteroid biosynthesis^45^. This is the causal gene of a classic rice dwarf mutant, *ebisu dwarf* (*d2*)^45^. This is the first report of natural variation associated with several of these loci, most of which were discovered using mutant analyses.

To determine whether the major genes on chromosomes 3 and 5 interact epistatically with other loci, we performed a covariate vs SNP interaction scan using SNP-3.16732086 (*GS3*) and SNP-5.5371749 (*GW5*) as covariates. We detected a significant interaction between the MS-SNP at *GW5* and several SNPs in a ∼100 kb region on chromosome 4 (*P*=1.86e−21), while no significant interactions with *GS3* were identified. The chromosome 4 region showed no significant effect on grain size when tested independently, only as an interaction. The minor allele frequency (MAF) for the most significant epistatic-SNP (SNP-4.4260961) is 10% across all subpopulations; it is essentially fixed in *aus*, *indica* and *temperate japonica* and segregates only in *tropical japonica* (MAF=24%). This suggests that both epistasis and differences in allele frequency contribute to the distinctive genetic architectures associated with grain length in the different subpopulations. The SNPs with significant *P* values for the *GW5* interaction define a region on chromosome 4 containing 14 genes. Among these, the most notable is a gene encoding a C2H2 zinc-finger transcription factor (LOC_Os04g08034; Supplementary Fig. 5). RNA-seq data indicate that this gene is expressed in inflorescences pre and post emergence, as well as the seed (embryo and endosperm; http://rice.plantbiology.msu.edu/cgi-bin/ORF_infopage.cgi?orf=LOC_Os04g08034). Further investigation on the potential impact of this gene (or region) on grain length is warranted.

### Harnessing the power of subpopulation-specific GWAS

**Figure 6:**
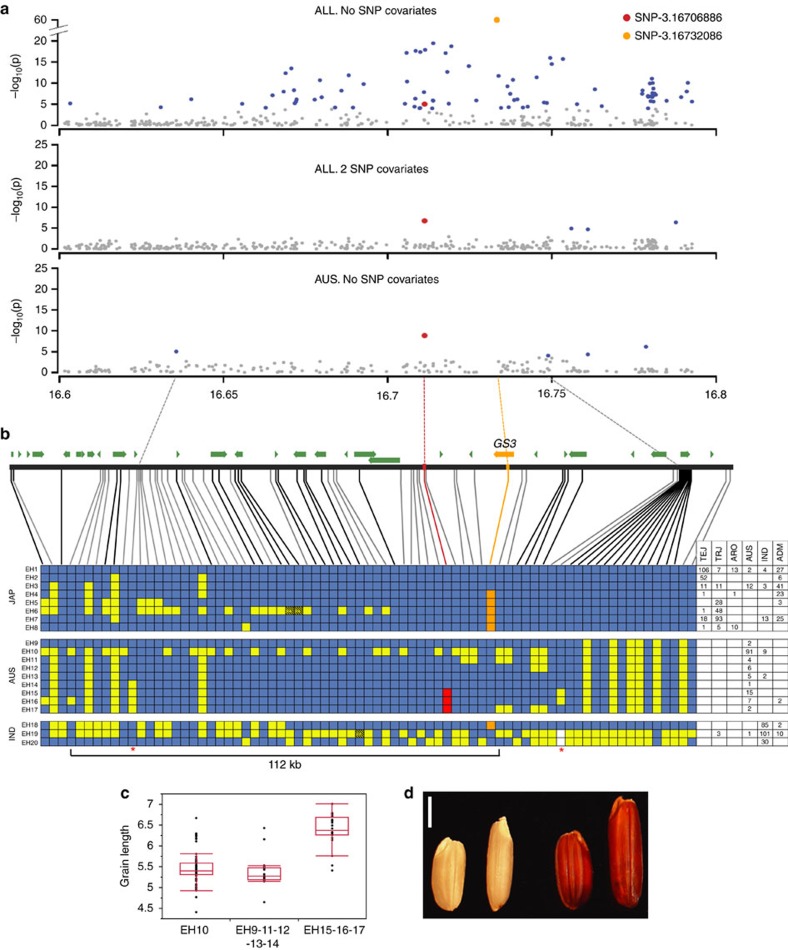
The *aus*-specific peak on chromosome 3. (**a**) Zoom-in of chromosome 3 (16.6–16.8 Mb) showing GWAS peaks over *GS3* using the HDRA with RDP1 and 2 *ALL* (top), *ALL* with 2 SNP covariates (centre), and the *aus* subpopulation (bottom). The *aus* MS-SNP (SNP-3.16709886) is indicated by a red circle and the *ALL* MS-SNP (SNP-3.16732086) by an orange circle. Dashed lines connecting (**a**,**b**) indicate the position of the *aus* MS-SNP (red), the *GS3* FNP (orange), and the second and third MS-SNPs in *aus* (grey). (**b**) Extended haplotype (EH) analysis across the peak region in RDP1 and 2. Top track shows the position of each SNP used in the analysis, connected by lines to the haplotype table below. Gene models are represented by green arrows; *GS3* by an orange arrow. SNPs connected by a grey line are located in intergenic regions; those connected by a black line fall within genes. Rows in the haplotype table correspond to consensus haplotype groups constructed based on HDRA SNP data. Blue cells represent SNPs identical to the Nipponbare reference; yellow cells represent variant alleles; dashed yellow cells represent heterozygotes and white represent missing data. The *aus-*specific MS-SNP is highlighted in red and the *GS3* FNP in orange. The second and third *aus* MS-SNPs are indicated by red stars under the haplotype table. The number and subpopulation identity of varieties carrying each haplotype are indicated in the table. EHs are represented as a consensus and minor variation is present within each group. For clarity, low-frequency recombinant haplotypes are not displayed (except for those *aus* recombinant haplotypes informative for the present analysis). (**c**) Quantile boxplots showing phenotypic distribution (average grain length) for varieties carrying the common *aus* haplotype, varieties with introgression carrying the reference allele at SNP-3.16709886 (EH9, 11, 12, 13 and 14); and with introgression carrying the derived allele at SNP-3.16709886 (EH15, 16 and 17). The ends of the each box represent the 75th and 25th quantiles (**d**) Representative *aus* accessions with short and long grain phenotypes: Left to right: Ghorbhai/NSFTV326 (EH10), Phudugey/NSFTV131 (EH17), Jamir/NSFTV328 (EH12) and PTB-30/NSFTV360 (EH15). Scale bar, 2 mm.

Further examination of the peaks in each subpopulation demonstrates the value of GWAS as a road map for gene discovery. Here we highlight an example where a clear peak in the *GS3* region was observed in the *aus* subpopulation (Supplementary Fig. 4). The MS-SNP in *aus* (SNP-3.16709886) is located 18 kb to the left of *GS3* (Fig. 6a), two gene models away in an intergenic region, while the previously reported functional polymorphism in exon 2 of *GS3* (SNP-3.16732086) is fixed for the wild-type allele in *aus*, and therefore not significant. The *aus* MS-SNP-3.16709886 is also significant in *ALL* and in joint *INDICA* when SNP-3.16732086 (the *GS3* FNP) is included as a covariate in the GWAS model (Fig. 6a, second panel; Fig. 5a,b). These results suggest that SNP-3.16709886 tags variation for grain length that is independent of the known *GS3* allele. The mutation in exon two of *GS3* confers long grain, while four additional mutations in *GS3* (all occurring in exon 5) confer shorter grain^34^,^36^. Interestingly, the derived allele at the *aus* MS-SNP is associated with longer grains (Fig. 6).

Our GWAS results point to a potentially novel source of variation in or near *GS3* in the *aus* subpopulation. To better characterize variation in the region we constructed extended haplotypes (EHs) based on HDRA genotypes for the 1,146 rice lines phenotyped for grain length (Fig. 6b). The EHs encompass a 190 kb region extending beyond the perimeter of the *aus*-specific peak as defined by its three MS-SNPs. Similar to what we have described previously^36^, three well-differentiated major haplotype groups are observed in the region: *japonica*, *aus* and *indica* (according to the subpopulation in which each occurs at highest frequency). The most common *temperate japonica* haplotypes, EH1 and EH2 (83%) carry the wild-type allele at SNP-3.16732086, the common *tropical japonica* haplotypes, EH7, EH6 and EH5 (87%) carry the derived (long grain) *GS3* allele (indicated in orange in Fig. 6b), while the *aromatic* accessions are almost evenly split between two haplotypes carrying the wild-type and derived alleles at *GS3* (EH1 and EH8, respectively). The mutation marked by SNP-3.16732086 is known to have occurred in a *japonica* background then introgressed into *indica* (EH7 and EH18), but it is notably rare in *aus* and was not observed in any of the *aus* varieties in our panel.

The common *aus* haplotype is EH10 (62%) and the common *indica* haplotype is EH19 (40.9%). Two *aus* accessions carry the common *japonica* haplotype EH1, 12 accessions carry EH3 and two carry the recombinant EH9 haplotype, while 40 additional *aus* varieties are distributed among seven *aus*-specific haplotypes: three with the derived allele at SNP-3.16709886 (EH15/16/17) and four with the *wt* allele (EH11–14). These seven haplotypes are similar to EH3, but are differentiated by specific recombination break points within the *aus* background. Varieties carrying EH15/16/17 have significantly longer grains than those carrying the common *aus* haplotype EH10 (Fig. 6c,d), and even more so than lines carrying EH9/11/12/13/14, which are genetically similar across the region.

All 24 *aus* varieties that carry the derived MS-SNP (SNP-3.16709886) also carry a second SNP (indicated by the left red star on Fig. 6b). One short-grained *aus* variety was found to carry the second MS-SNP but not the derived allele at SNP-3.16709886 (EH14). The third MS-SNP (indicated by the right red star on Fig. 6b) is present in all 22 *aus* varieties carrying EH15/16, but not in the 2 lines carrying EH17. The recombination break points in EH16 on the left and in EH17 on the right allow us to define a 112-kb candidate region underlying this novel association with grain length (indicated by a bracket in Fig. 6b). The target region contains 13 gene models and includes *GS3* and its regulatory region. Additional random mutations across the *japonica-*like region of introgression are observed in many of the *aus* lines documented here (Supplementary Data 5). None of these mutations are informative in the context of this analysis, though they do suggest that the introgression is not a recent event.

Lastly, it is interesting to note that there is significant variation for grain length among the varieties carrying the common *aus* haplotype, EH10. This variation is the source of the minor GWAS-QTLs detected when SNP-3.16709886 is used as a covariate in the *aus* subpopulation-specific GWAS (Supplementary Fig. 4, Supplementary Data 3).

## Discussion

In this study, we set out to design a publicly available GWAS mapping platform that comprises genotype information on a set of accessions that will help to understand and dissect the genetic architecture of economically important traits in this crucial crop. We purified a total of 1,568 rice accessions and developed a 700,000-SNP array (HDRA) that assays one SNP approximately every 0.54 kb across the rice genome and is enriched for SNPs in and around genes.

In terms of SNP density, the HDRA offers more SNPs per kb than the highest density commercial human genotyping array (Illumina Human OMNI 5-4 BeadChips; Illumina, San Diego, CA, USA) and surpasses the largest publicly available genotyping platform in any crop species, namely a maize array with 600,000 SNPs, or 1 SNP per 4 kb (ref. 59). While array technology has represented the gold standard in genotyping for many years, and is advantageous for generating high-quality data and efficiently processing large numbers of samples, it has inherent ascertainment bias owing to the emphasis on common SNPs in the discovery panel. While the decreasing cost and continued technical improvements in the throughput and quality of next-generation sequencing platforms is rapidly displacing the use of fixed arrays for genotyping, high-quality array data, such as that reported here, provides a valuable framework of diversity information into which new re-sequencing information can be readily integrated. In keeping with this potential, the recent re-sequencing of several thousand rice genomes^5^,^8^ promises to enhance the utility of the resources and analysis tools described in this study. Though the rice varieties sequenced to date do not overlap significantly with the RDP1 and 2 plant materials reported here, haplotype information derived from the re-sequencing data can be readily integrated with the HDRA SNP data set to enhance the resolution of the genotypic data set, providing new opportunities to identify candidate genes and functional polymorphisms underlying many of the QTLs identified by GWAS.

The ability to identify both large- and small-effect QTL associated with a phenotype of interest is of great interest to breeders. By including the most significant SNP(s) from each experiment as a covariate(s) in the GWA model, we demonstrate our ability to identify both common and subpopulation-specific alleles. The high heritability of grain length suggests that this trait may be conditioned by only a few genes of large effect, but the range of variation for grain size detected in this collection of germplasm (4.2–7.3 mm) and the combination of both natural and artificial selection that have influenced trait expression in different subpopulations and environments, hint at greater complexity. Here, we demonstrate that, in addition to well-characterized genes of large effect, our approach allows for the detection of numerous genetic factors of small effect that contribute to the highly valued grain quality characteristics that distinguish local rice varieties, supported by the fact that several peaks co-localize with known QTL regions and candidate genes related to grain shape and/or size. In cases where well-characterized grain size genes do not co-locate with GWAS peaks, it is presumably because the functional alleles at these loci occur at low frequency in our diversity panel, or have immeasurably small allele effects in the context of other loci segregating for grain length. GWAS peaks identified in regions where no QTL for grain size has been mapped to date provide new opportunities for gene discovery.

Grain length is one of many traits for which different cultures and commercial markets show strong and often opposing preferences^58^. In Japan, Korea and northern China, short grain rice is strongly preferred. These areas grow almost exclusively *temperate japonica* varieties that have near universally short, wide grains and are adapted to cooler seasons and the long days of higher latitudes. Long, slender grains are preferred in South Asia and parts of South East Asia where *indica* varieties are grown; short, thin grains are prized in traditional *aus-*growing regions such as Bangladesh and southeastern parts of India, and very long, thin, aromatic grains are the specialty of the *basmati* and *sadri-*growing provinces of India, Pakistan, Afganistan and Iran^58^,^60^. Knowledge about specific genes and alleles that contribute to grain quality and yield in different genetic backgrounds will assist rice breeders in fine-tuning their selection for superior grain quality, and has implications for other crops where grain morphology is also highly valued.

In a companion paper by Crowell *et al.*^24^, we explore the potential to use the rice diversity panel and accompanying HDRA SNP data set to dissect the genetics of panicle architecture on field-grown plants using a high-resolution imaging system as the basis for phenotyping. We demonstrate the potential to build ‘association networks' based on GWAS analysis, and to use phenotypic covariates to dissect multi-trait GWA-peaks, with direct implications for applied plant breeding.

**Figure 7:**
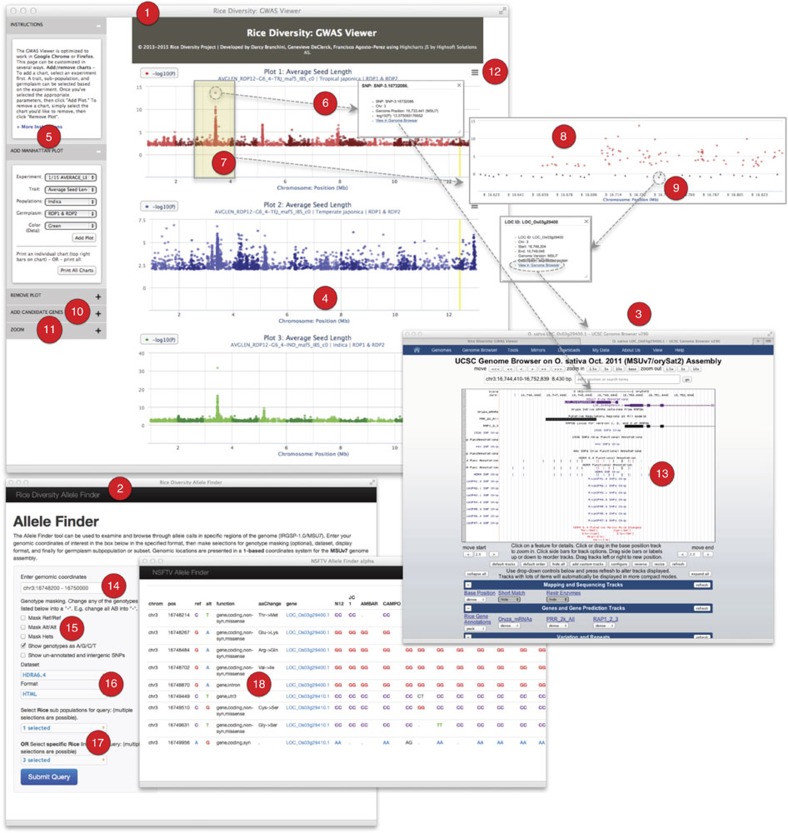
Data visualization tools. The GWAS Viewer (1) and the Allele Finder (2) were developed by the Rice Diversity Project to facilitate interpretation of GWAS results and the HDRA genotype data set. A local instance of the UCSC Genome Browser (3) was also created. All are available for public use at www.ricediversity.org. Users can select up to six plots to display in the main GWAS Viewer pane (4) by making selections in the control panel at the left (5). Points display SNP ID, position and *P* value when moused-over. Clicking on a data point (6) provides similar information in a pop-up window, along with a hyperlink to the Genome Browser. Users can highlight and zoom-in on a particular region by holding a click and dragging over (7,8) the region. When zoomed-in the IRGSP-1.0/MSU7 genome annotation features are displayed as grey triangles (9) and can be moused-over or clicked on for more information. Users can enter up to 100 MSU7 LOC IDs in the ‘Add Candidate Genes' box (10) for display on a selected plot as orange triangles. A synchronized-zoom feature is available (11). Print or download an image by clicking at the top right of the plot (12), or click on the ‘Print All Charts' button to print or download all plots displayed in the current session. Our local instance of the UCSC Genome Browser has several tracks of data (13) in addition to the IRGSP-1.0/MSU7 annotation; these include the RAP-DB genome annotation^56^, positions of SNPs on the Rice Diversity HDRA, the 44 K (ref. 17) and 1,536 (ref. 57) SNP chips and their functional annotations, and SNP sets from the Rice OPA breeder's mini-chips^52^. The Allele Finder tool allows the user to quickly find, examine and interpret specific regions of HDRA genotype data set. Enter a genome region of interest (14), select masking and display options (15), a data set and output format (16), select germplasm groups or subpopulations (17), and click the submit button. Along with the allele data the query results (18) display position information, reference and alternate allele, functional information, LOC_Os IDs, and predicted amino acid changes.

In addition to the SNP data set, genetic resources and analytical strategies presented here, we have also developed a suite of bioinformatics tools to facilitate the analysis of GWAS data (Fig. 7, Supplementary Note 2, Supplementary Software 1). These tools are comprised of a GWAS Viewer, Allele Finder and Genome Browser. The GWAS Viewer is a web-based interactive tool for visualization and analysis of GWAS data; the Allele Finder is a tool for querying and retrieving large genotype data sets in a user-friendly graphical interface; and our local mirror of the UCSC Genome Browser facilitates the analysis of HDRA and other SNP data sets in a genomic context. Together, these resources represent an open-source research platform that will empower the plant community to explore the genetics underlying a wide array of complex traits, providing new opportunities for geneticists and breeders to collectively understand and better utilize the wealth of natural variation that is conserved in gene banks around the world.

## Methods

### Germplasm

The germplasm collections from which accessions used in this study were drawn are summarized in Supplementary Data 1. They consist of three different collections: (i) the RDP1 representing 433 accessions (423 accessions with seed for distribution) from 78 different countries, as described previously^16^,^33^; (ii) the RDP2 representing 1,445 accessions from 92 different countries, described here for the first time; and (iii) accessions contributed by The National Institute of Agrobiological Sciences (NIAS)^61^,^62^,^63^ consisting of 75 accessions from 19 different countries. The subpopulation distribution of lines analyzed for GWAS differed for the three diversity panels. Briefly, RDP1 consisted of 20% *indica*, 13% *aus*, 25% *temperate japonica*, 25% *tropical japonica*, 3% *aromatic* and 18% *admixed* accessions; the RDP2 consisted of 35% *indica*, 11% *aus*, 12% *temperate japonica*, 23% *tropical japonica*, 2% *aromatic* and 17% *admixed* accessions; the NIAS panel consisted of 39% *indica*, 23% *aus*, 15% *temperate japonica*, 5% *tropical japonica*, 0% *aromatic* and 18% *admixed* accessions (Supplementary Data 1,2).

Seeds were originally obtained from (i) the International Rice Genebank Collection at the International Rice Research Institute (IRRI) in the Philippines (http://irri.org/our-work/seeds), (ii) the USDA-ARS National Small Grains Collection (NSGC), Aberdeen, ID, USA, which is part of the National Plant Germplasm System (www.ars-grin.gov/npgs) and (iii) the NIAS Genebank in the National Germplasm Collection in Tsukuba, Japan (http://www.gene.affrc.go.jp). Because of the observed heterogeneity in the gene bank accessions, all materials were purified for two generations via single seed descent before genotyping. The purified lines analyzed here can be considered representative haplotypes from the original, heterogeneous landrace accessions available in the gene banks.

### The collection of varieties

To acknowledge their unique genetic composition, RDP1 and RDP2 materials are registered as genetic stocks and have been assigned new accession numbers in the T.T. Chang Genetic Resources Center at IRRI, and in the Genetic Stocks-*Oryza* collection at the Dale Bumpers National Research Center in Stuttgart, AR, USA. Information about RDP1 accessions can be found in Eizenga *et al.*^33^. RDP2 accessions were selected to increase the number of accessions available for GWA, limiting the degree of potential duplication, and simultaneously to better represent the range of genetic variation in the combined RDP1 and 2 panels. In compiling the RDP2, particular emphasis was placed on the inclusion of *O. sativa* varieties adapted to parts of the world where food security is most at risk, as well as those that are or have been widely used in genetic studies and breeding at IRRI and among its national program partners. See Supplementary Data 1 for a detailed list of accessions, including genebank accession IDs, variety/common name, country of origin and subpopulation identity based on FastSTRUCTURE analysis.

### Development of the HDRA

Methods for the development of the HDRA, including SNP discovery and selection, probe design, probe preparation and hybridization, genotype calling and quality control can be found in Supplementary Note 1.

### SNP functional annotation

A total of 66,152 gene models were downloaded from the MSU Rice Genome Annotation Project version 7 in GFF3 format. Data were converted to the UCSC Browser's BED format and then uploaded to a local instance of the Galaxy Project bioinformatics server (http://galaxyproject.org/). All locations of exons, introns and UTR regions from the MSUv7 gene annotation were extracted using Galaxy's *Gene BED To Exon/Intron/Codon* tool. Assignment of SNPs to the extracted genomic features was determined by detecting overlaps with each feature's locus using the UCSC Browser's liftOver tool (https://genome.ucsc.edu/util.html). For those SNPs that overlapped an exon, further analysis was done using Galaxy's Amino Acid changes (aaChanges) tool in order to determine the character (for example, synonymous or non-synonymous) of each SNP's induced mutation. A script written in Python was used to merge and integrate all the results from the previous steps and assign each HDRA SNP to one or more functional categories. SNPs were classified as UTR5, UTR3, exonic, intronic based on overlap with the regions of the same name. In addition, SNPs were assigned to PPRs defined as segments within 2 kb upstream from the transcription start site. SNPs overlapping the PPRs were determined as described above. Since many SNPs were associated with more than one putative function, precedence was established for assigning SNPs to a primary category for counting purposes. The primary category was assigned using a Python script that assigned SNPs in the following priority order: exonic, intronic, UTR (5′/3′), PPR. The primary category assignment for each SNP was used as the basis for the rest of the functional analyses.

### Principal components analysis

PCs were extracted from the genetic correlation matrix (using only SNPs present in all lines) by applying the svd function in the R software package^64^.

### Site frequency spectra

To generate site-frequency spectra, for synonymous and non-synonymous SNPs we divided the SNPs into 100 frequency intervals and counted the number of SNPs in each bin. We did this separately for synonymous and non-synonymous SNPs to compare the distributions within each category, making sure that binning frequency was the same and thus yielded comparable spectra. We made these spectra for RDP1 and 2 (*ALL*) as well as for *INDICA* and *JAPONICA* varietal groups. Since we do not have an outgroup, we plotted only minor allele frequencies, thus the frequency values do not exceed 0.5 on the plots (Supplementary Fig. 2). We also tried a range of bin numbers from 50 to 500, with the same results.

### LD Decay

To assess LD among SNP markers in each of the *O. sativa* subpopulations (*aus*, *indica*, *tropical japonica*, *temperate japonica*, *aromatic*), we identified lines that belonged unambiguously to each group based on FastSTRUCTURE results (>70% ancestry). We discarded SNPs with >33% missing data and with fewer than three individuals carrying the minor allele. The samples consisted of 469 individuals (with 536,769 SNP markers) from *indica*; 170 individuals (430,077 markers) from *aus*; 356 lines (443,570 markers) from *tropical japonica*; and 229 lines (313,378 markers) from *temperate japonica*. We moved 4,000-SNP windows along each chromosome. In each window, we calculated LD between all pairs of markers, using only lines with pairwise complete data. As measures of LD, we calculated *r*^*2*^ (the square of the correlation coefficient between SNP states) and the absolute value of *D'* (Lewontin, 1964)^65^. The latter measure is more robust to allele frequency fluctuations than *r*^*2*^. As we calculated LD, we noted the distances between SNP markers. We then assigned the distances to 500 bins and calculated mean LD within each band of distances.

### Phenotyping for grain length

Three replicates of the complete set of samples were grown in the 2011 wet season in IRRI fields in the Philippines, using standard crop management practices for irrigation and pest control. A subset of 129 accessions that spanned the range in grain lengths observed during 2011 was also grown during the dry season of 2012 at IRRI and grain length measured on these varieties was used to estimate broad sense heritability^66^ using the following equation:


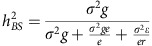


where 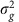 is the genotypic variance, 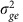 is the genotype by environment interaction variance, *σ*^2^ is the experimental error variance, *r* is replicates and *e* is environments. For both the full set (2011) and the subset (2012), grain was harvested at maturity and stored for 6 weeks under controlled relative humidity, to equilibrate for moisture content. A sample of paddy (10 g) was dehulled (THU35A 250V 50Hz Test Husker, Satake), and polished by placing grains into 10 ml capsules with fused aluminium oxide and then abrading gently for 1 h in a paint shaker. Grain length was measured on an average of 75 individual grains from each of three biological replicates, with a Cervitec Grain Inspector 1625 (FOSS Analytical, Hoganas, Sweden; Calingacion *et al.*^60^) at the Grain Quality and Nutrition Center at IRRI.

### Genome-wide association studies

GWAS were run using a linear-mixed model in EMMAX vs beta-07Mar2010 (ref. 67), which takes underlying population structure into account by including a kinship matrix as a covariate. The germplasm samples used for generating the HDRA SNP data set and all resulting genotyping GWA results were grown in 2011, and are subsets of the RDP collections consisting of 406 accessions from RDP1 and 1,087 from RDP2 (Supplementary Data 1). The genotype data was limited to include only lines with phenotypes for the trait of interest (average seed length), and SNPs with MAF >0.05 in each subpopulation, as well as at least 3 minor allele count and 30% maximum missing data. These lines were included in the calculation of the kinship matrix. When the analysis was run across all available lines in the panel, SNPs present at MAF>0.05 in individual subpopulations were combined and three additional PC covariates were added to the model. No PC covariates were used in analyses within subpopulations (*indica*, *aus*, *temperate japonica* and *tropical japonica*). A significance threshold of 10% false discovery rate (R implementation of Benjamini and Hochberg^68^) was calculated. A GWAS pipeline for matching germplasm data and phenotypes, filtering and farther data processing was built in Python 2.6 using R scripts for making plots, and is available for download and use at ricediversity.org.

### Allele effects, percent variation explained and epistasis scans

To calculate PVE and report this for all tests in the EMMAX results, we modified EMMAX C source code to perform this calculation using the model term variance that EMMAX already extracts to conduct the *t*-test for significance and report *P*values for the tested marker. We also modified EMMAX to calculate the total per cent variance explained of the model (including the random effects term) for every test as well as the additive genetic variance explained (without the random effects term). For total fixed effect genetic variance explained (relevant when two or more marker terms are included—see below), we extracted the residuals from an initial model fit with dummy marker terms substituted with random genotypes to produce the same degrees of freedom as each model of interest and calculated the variance. We subtracted this from the phenotypic variance to get the variance explained by the kinship matrix random effects term. In the model tests including fixed genetic effect terms, we subtracted this value from the total model variance explained to obtain the variance explained by the additive genetic effects of the markers. In the additive linear mixed model genotype variables were coded as AA=−1, AB=0, BB=1. Allele effects correspond to the regression coefficient. This is the expected absolute change in grain length for each B (non-reference) allele with twice this value equal to the expected grain length difference between the BB and AA homozygotes. This corresponds to the standard additive effects quantitative genetic model, where the dominance deviation *d* is constrained to be *d*=0 (co-dominant locus).

To perform a GWAS analysis for possible epistatic interaction effects with SNPs detected as highly significant in our single-marker scans, we modified further modified the EMMAX to allow specific markers to be specified on the command line as fixed model covariates. The modified EMMAX would then select these markers out of the input data and include the genotypes at these SNPs (more than one may be specified) as additive fixed-effect independent model terms in addition to markers being tested one-by-one in the genome-wide scan (the ‘scan marker'). Allele effect size, *P* value and PVE were reported for these terms in every test as well as these values for the scan marker. As expected, fixed terms specified in this way generated similar allele effects, *P* values and PVEs as they did in their single marker test in the initial GWAS scan. Finally, we tested interactions of scan markers with these fixed covariate terms by modifying EMMAX source to calculate an epistatic interaction term as −1 for AA/AA, 0 for AA/BB and BB/AA and +1 for BB/BB; and 0 for any combination including a heterozygote genotype. This interaction term, as well as the independent effect term(s) for the specified markers, was inserted into the mixed-model fixed effect matrix (along with the scan marker). As with the independent covariate SNP terms, interaction effect size, *P* value and PVEs were output for all genome-wide tests.

### Haplotype analysis across *GS3* region

EHs spanning a 190 kb region flanking *GS3* and the *aus*-specific peak in the region were constructed on the 1,146 *O. sativa* accessions from RDP1 and 2 genotyped with the HDRA and phenotyped for grain length. The HDRA carries a total of 424 total SNPs in the 190 kb region. SNPs with a MAF>0.05 and <3% missing data were initially selected. SNPs were then filtered based on a frequency test; only SNPs with a significant frequency difference between the *INDICA* and *JAPONICA* varietal groups (*P* value cutoff of 1.0e−06). The final set of SNPs used in constructing the haplotype map in Fig. 6 consisted of 76 SNPs. The complete SNP data set for the region is available in Supplementary Data 5.

## Additional information

**Accession codes:** SNP information has been deposited in the NCBI dbSNP database (batch ID 1062024) and the GEO database (accession ID: GSE71553).

**How to cite this article:** McCouch, S. R. *et al.* Open access resources for genome-wide association mapping in rice. *Nat. Commun.* 7:10532 doi: 10.1038/ncomms10532 (2016).

## Supplementary Material

Supplementary Figures, Supplementary Tables and Supplementary References Supplementary Figure 1-5, Supplementary Tables 1-2 and Supplementary References

Supplementary Data 1 RDP1&2 germplasm collection. “This table presents the entire RDP1 and RDP2 collections, a subset of which (denoted with the presence of a HDRA assay id in the third column) were genotyped using the HDRA. Detailed column descriptors can be found under the table.

Supplementary Data 2 Summary of SNP polymorphisms for each rice diversity panel and subpopulation

Supplementary Data 3 P values, allele effects and percent variance explained (PVE) for the most significant SNP in each GWAS peak identified with and without SNP covariates

Supplementary Data 4 List of candidate genes, loci and QTL regions mapped for grain length, weight, or other grain shape traits, included in our analysis

Supplementary Data 5 Raw genotype data (HDRA SNPs) in the region of the aus-specific peak. This was the raw data used in the construction of the haplotype map in Figure 6.

Supplementary Software 1 GWAS Pipeline

## Figures and Tables

**Table 1:** Summary of polymorphisms detected by the HDRA in the different rice subpopulations.

	***O. sativa***	**IND**	**AUS**	**TRJ**	**TEJ**	**ARO**
Total accessions	1,571	498	187	360	240	38
Phenotyped accessions*	1,146	335	160	256	190	31
Polymorphic SNPs	671,906	619,988	547,928	551,746	470,318	392,943
Non-synonymous polymorphic SNPs	109,814	100,762	88,093	89,863	78,259	62,560
SNPs MAF>0.05	439,280	336,740	328,393	208,055	170,904	290,068
SNPs MAF>0.10	300,769	223,094	238,308	120,538	94,826	195,271
Private SNPs†	—	25,718	10,961	9,322	4,916	3,383

MAF, major allele frequency; SNP, single-nucleotide polymorphism.

^*^Accessions phenotyped for grain length.

^†^Private SNPs are the SNPs segregating only in the indicated subpopulation.

## References

[b1] FAO. Increasing Crop Production Sustainably. The Perspective of Biological Processes Food and Agriculture Organization of the United Nations, Rome (2009).

[b2] Bouman B. A. M., Barker R., Humphreys E. & Tuong T. P. in Water for Food, Water for Life ed. Molden D. Earthscan; International Water Management Institute (2007).

[b3] Jackson M. T. Conservation of rice genetic resources: the role of the International Rice Genebank at IRRI. Plant Mol. Biol. 35, 61–67 (1997). 9291960

[b4] International Rice Genome Sequencing Project. The map-based sequence of the rice genome. Nature 436, 793–800 (2005). 16100779 10.1038/nature03895

[b5] The rice genome project. The 3,000 rice genomes project. Gigascience 3, 7 (2014). 24872877 10.1186/2047-217X-3-7 PMC4035669

[b6] Huang X. *et al.* A map of rice genome variation reveals the origin of cultivated rice. Nature 490, 497–501 (2012). 23034647 10.1038/nature11532 PMC7518720

[b7] Xu X. *et al.* Resequencing 50 accessions of cultivated and wild rice yields markers for identifying agronomically important genes. Nat. Biotechnol. 30, 105–111 (2012). 22158310 10.1038/nbt.2050

[b8] Duitama J. *et al.* Whole genome sequencing of elite rice cultivars as a comprehensive information resource for marker assisted selection. PLoS ONE 10, e0124617 (2015). 25923345 10.1371/journal.pone.0124617 PMC4414565

[b9] Schatz M. *et al.* Whole genome de novo assemblies of three divergent strains of rice, *Oryza sativa*, document novel gene space of *aus* and *indica*. Genome Biol. 15, 506 (2014). 25468217 10.1186/s13059-014-0506-z PMC4268812

[b10] Sakai H. *et al.* Construction of pseudomolecule sequences of the aus rice cultivar Kasalath for comparative genomics of asian cultivated rice. DNA Res. 21, 397–405 (2014). 24578372 10.1093/dnares/dsu006 PMC4131834

[b11] Yu J. *et al.* A draft sequence of the rice genome (*Oryza sativa* L. ssp. *indica*). Science 296, 79–92 (2002). 11935017 10.1126/science.1068037

[b12] Jacquemin J., Bhatia D., Singh K. & Wing R. A. The International Oryza Map Alignment Project: development of a genus-wide comparative genomics platform to help solve the 9 billion-people question. Curr. Opin. Plant Biol. 16, 147–156 (2013). 23518283 10.1016/j.pbi.2013.02.014

[b13] Famoso A. N. *et al.* Genetic architecture of aluminum tolerance in rice (*Oryza sativa*) determined through genome-wide association analysis and QTL mapping. PLoS Genet 7, e1002221 (2011). 21829395 10.1371/journal.pgen.1002221 PMC3150440

[b14] Huang X. *et al.* Genome-wide association studies of 14 agronomic traits in rice landraces. Nat. Genet. 42, 961–967 (2010). 20972439 10.1038/ng.695

[b15] Huang X. *et al.* Genome-wide association study of flowering time and grain yield traits in a worldwide collection of rice germplasm. Nat. Genet. 44, 32–39 (2012). 22138690 10.1038/ng.1018

[b16] Zhao K. *et al.* Genome-wide association mapping reveals a rich genetic architecture of complex traits in *Oryza sativa*. Nat. Commun. 2, 467 (2011). 21915109 10.1038/ncomms1467 PMC3195253

[b17] Shan Q., Wang Y., Li J. & Gao C. Genome editing in rice and wheat using the CRISPR/Cas system. Nat. Protoc. 9, 2395–2410 (2014). 25232936 10.1038/nprot.2014.157

[b18] Miao J. *et al.* Targeted mutagenesis in rice using CRISPR-Cas system. Cell Res. 23, 1233–1236 (2013). 23999856 10.1038/cr.2013.123 PMC3790239

[b19] Zhou H., Liu B., Weeks D. P., Spalding M. H. & Yang B. Large chromosomal deletions and heritable small genetic changes induced by CRISPR/Cas9 in rice. Nucleic Acids Res. 42, 10903–10914 (2014). 25200087 10.1093/nar/gku806 PMC4176183

[b20] Collard B. C. & Mackill D. J. Marker-assisted selection: an approach for precision plant breeding in the twenty-first century. Philos. Trans. R. Soc. Lond. B Biol. Sci. 363, 557–572 (2008). 17715053 10.1098/rstb.2007.2170 PMC2610170

[b21] Septiningsih E. M. *et al.* Development of submergence-tolerant rice cultivars: the Sub1 locus and beyond. Ann. Bot. 103, 151–160 (2009). 18974101 10.1093/aob/mcn206 PMC2707316

[b22] Jiang G.-L. Molecular Markers and Marker-Assisted Breeding in Plants, Plant Breeding from Laboratories to Fields ed. Andersen S. B. DOI: 10.5772/52583 InTech (2013).

[b23] Shigesaburo T. & Takahashi N. in Developments in Crop Science Vol. 7ii, Elsevier (1984).

[b24] Crowell S. *et al.* Genome-wide association and high-resolution phenotyping link *Oryza sativa* panicle traits to numerous trait-specific QTL clusters. Nat. Commun. 7, 10527 (2015). 26841834 10.1038/ncomms10527 PMC4742901

[b25] Zhao Y., Mette M. F., Gowda M., Longin C. F. H. & Reif J. C. Bridging the gap between marker-assisted and genomic selection of heading time and plant height in hybrid wheat. Heredity 112, 638–645 (2014). 24518889 10.1038/hdy.2014.1 PMC4023446

[b26] Zhang Z. *et al.* Improving the accuracy of whole genome prediction for complex traits using the results of genome wide association studies. PLoS ONE 9, e93017 (2014). 24663104 10.1371/journal.pone.0093017 PMC3963961

[b27] Spindel J. E. *et al.* Genome-wide prediction models that incorporate *de novo* GWAS are a powerful new tool for tropical rice improvement. Heredity (in press). 10.1038/hdy.2015.113 PMC4806696 26860200

[b28] Raj A., Stephens M. & Pritchard J. K. fastSTRUCTURE: variational inference of population structure in large SNP data sets. Genetics 197, 573–589 (2014). 24700103 10.1534/genetics.114.164350 PMC4063916

[b29] Garris A. J., Tai T. H., Coburn J., Kresovich S. & McCouch S. Genetic structure and diversity in *Oryza sativa* L. Genetics 169, 1631–1638 (2005). 15654106 10.1534/genetics.104.035642 PMC1449546

[b30] Xu Y. *et al.* Leaf-level water use efficiency determined by carbon isotope discrimination in rice seedlings: genetic variation associated with population structure and QTL mapping. Theor. Appl. Genet. 118, 1065–1081 (2009). 19224195 10.1007/s00122-009-0963-z

[b31] Price A. L. *et al.* Principal components analysis corrects for stratification in genome-wide association studies. Nat. Genet. 38, 904–909 (2006). 16862161 10.1038/ng1847

[b32] Kojima Y., Ebana K., Fukuoka S., Nagamine T. & Kawase M. Development of an RFLP-based rice diversity research set of germplasm. Breed Sci. 55, 431–440 (2005).

[b33] Eizenga G. C. *et al.* Registration of the rice diversity panel 1 for genomewide association studies. J. Plant Reg. 8, 109–116 (2014).

[b34] Takano-Kai N. *et al.* Evolutionary history of GS3, a gene conferring grain length in rice. Genetics 182, 1323–1334 (2009). 19506305 10.1534/genetics.109.103002 PMC2728869

[b35] Fan C. *et al.* GS3, a major QTL for grain length and weight and minor QTL for grain width and thickness in rice, encodes a putative transmembrane protein. Theor. Appl. Genet. 112, 1164–1171 (2006). 16453132 10.1007/s00122-006-0218-1

[b36] Takano-Kai N. *et al.* Multiple and independent origins of short seeded alleles of *GS3* in rice. Breed. Sci. 63, 77–85 (2013). 23641184 10.1270/jsbbs.63.77 PMC3621448

[b37] Wang C., Chen S. & Yu S. Functional markers developed from multiple loci in GS3 for fine marker-assisted selection of grain length in rice. Theor. Appl. Genet. 122, 905–913 (2011). 21107518 10.1007/s00122-010-1497-0

[b38] Shomura A. *et al.* Deletion in a gene associated with grain size increased yields during rice domestication. Nat. Genet. 40, 1023–1028 (2008). 18604208 10.1038/ng.169

[b39] Li Y. *et al.* Natural variation in GS5 plays an important role in regulating grain size and yield in rice. Nat. Genet. 43, 1266–1269 (2011). 22019783 10.1038/ng.977

[b40] Weng J. *et al.* Isolation and initial characterization of GW5, a major QTL associated with rice grain width and weight. Cell Res. 18, 1199–1209 (2008). 19015668 10.1038/cr.2008.307

[b41] Huang R. *et al.* Genetic bases of rice grain shape: so many genes, so little known. Trends Plant Sci. 18, 218–226 (2013). 23218902 10.1016/j.tplants.2012.11.001

[b42] Wang S. *et al.* Control of grain size, shape and quality by OsSPL16 in rice. Nat. Genet. 44, 950–954 (2012). 22729225 10.1038/ng.2327

[b43] Bai X. *et al.* Genetic dissection of rice grain shape using a recombinant inbred line population derived from two contrasting parents and fine mapping a pleiotropic quantitative trait locus qGL7. BMC Genet. 11, 16 (2010). 20184774 10.1186/1471-2156-11-16 PMC2846863

[b44] Kitagawa K. *et al.* A novel kinesin 13 protein regulating rice seed length. Plant Cell Physiol. 51, 1315–1329 (2010). 20587735 10.1093/pcp/pcq092

[b45] Hong Z. *et al.* A rice brassinosteroid-deficient mutant, ebisu dwarf (d2), is caused by a loss of function of a new member of cytochrome P450. Plant Cell 15, 2900–2910 (2003). 14615594 10.1105/tpc.014712 PMC282825

[b46] Ashikari M., Wu J., Yano M., Sasaki T. & Yoshimura A. Rice gibberellin-insensitive dwarf mutant gene Dwarf 1 encodes the α-subunit of GTP-binding protein. Proc. Natl Acad. Sci. 96, 10284–10289 (1999). 10468600 10.1073/pnas.96.18.10284 PMC17880

[b47] Tanabe S. *et al.* A novel cytochrome P450 is implicated in brassinosteroid biosynthesis via the characterization of a rice dwarf mutant, dwarf11, with reduced seed length. Plant Cell 17, 776–790 (2005). 15705958 10.1105/tpc.104.024950 PMC1069698

[b48] Song X.-J., Huang W., Shi M., Zhu M.-Z. & Lin H.-X. A QTL for rice grain width and weight encodes a previously unknown RING-type E3 ubiquitin ligase. Nat. Genet. 39, 623–630 (2007). 17417637 10.1038/ng2014

[b49] Yamamuro C. *et al.* Loss of function of a rice brassinosteroid insensitive1 homolog prevents internode elongation and bending of the lamina joint. Plant Cell 12, 1591–1605 (2000). 11006334 10.1105/tpc.12.9.1591 PMC149072

[b50] Wang E. *et al.* Control of rice grain-filling and yield by a gene with a potential signature of domestication. Nat. Genet. 40, 1370–1374 (2008). 18820698 10.1038/ng.220

[b51] Abe Y. *et al.* The *SMALL AND ROUND SEED1* (*SRS1/DEP2*) gene is involved in the regulation of seed size in rice. Genes Genet. Syst. 85, 327–339 (2010). 21317545 10.1266/ggs.85.327

[b52] Segami S. *et al.* Small and round seed 5 gene encodes alpha-tubulin regulating seed cell elongation in rice. Rice 5, 4–4 (2012). 24764504 10.1186/1939-8433-5-4 PMC3834490

[b53] Wu T. *et al.* Gene SGL, encoding a kinesin-like protein with transactivation activity, is involved in grain length and plant height in rice. Plant Cell Rep. 33, 235–244 (2014). 24170341 10.1007/s00299-013-1524-0

[b54] Wang Y. *et al.* Copy number variation at the GL7 locus contributes to grain size diversity in rice. Nat. Genet. 47, 944–948 (2015). 26147619 10.1038/ng.3346

[b55] Wang S. *et al.* The OsSPL16-GW7 regulatory module determines grain shape and simultaneously improves rice yield and grain quality. Nat. Genet. 47, 949–954 (2015). 26147620 10.1038/ng.3352

[b56] Heang D. & Sassa H. An atypical bHLH protein encoded by POSITIVE REGULATOR OF GRAIN LENGTH 2 is involved in controlling grain length and weight of rice through interaction with a typical bHLH protein APG. Breed. Sci. 62, 133–141 (2012). 23136524 10.1270/jsbbs.62.133 PMC3405970

[b57] Heang D. & Sassa H. Antagonistic actions of HLH/bHLH proteins are involved in grain length and weight in rice. Plos ONE 7, e31325 (2012). 22363621 10.1371/journal.pone.0031325 PMC3283642

[b58] Fitzgerald M. A., McCouch S. R. & Hall R. D. Not just a grain of rice: the quest for quality. Trends Plant Sci. 14, 133–139 (2009). 19230745 10.1016/j.tplants.2008.12.004

[b59] Unterseer S. *et al.* A powerful tool for genome analysis in maize: development and evaluation of the high density 600 k SNP genotyping array. BMC Genomics 15, 823 (2014). 25266061 10.1186/1471-2164-15-823 PMC4192734

[b60] Calingacion M. *et al.* Diversity of global rice markets and the science required for consumer-targeted rice breeding. PLoS ONE 9, e85106 (2014). 24454799 10.1371/journal.pone.0085106 PMC3893639

[b61] Ebana K., Kojima Y., Fukuoka S., Nagamine T. & Kawase M. Development of mini core collection of Japanese rice landrace. Breed. Sci. 58, 281–291 (2008).

[b62] Yamamoto T. *et al.* Fine definition of the pedigree haplotypes of closely related rice cultivars by means of genome-wide discovery of single-nucleotide polymorphisms. BMC Genomics 11, 267 (2010). 20423466 10.1186/1471-2164-11-267 PMC2874813

[b63] Yonemaru J. *et al.* Genome-wide haplotype changes produced by artificial selection during modern rice breeding in Japan. PLoS ONE 7, e32982 (2012). 22427922 10.1371/journal.pone.0032982 PMC3302797

[b64] The R Core Team. R: A Language and Environment for Statistical Computing http://www.R-project.org (2014).

[b65] Lewontin R. C. The interaction of selection and linkage. I. General considerations; heterotic models. Genetics 49, 49–67 (1964). 17248194 10.1093/genetics/49.1.49 PMC1210557

[b66] Hallauer A. R. & Fo J. B. M. Quantitative Genetics in Maize Breeding 2nd edn Iowa State University (1988).

[b67] Zhang Z. *et al.* Mixed linear model approach adapted for genome-wide association studies. Nat. Genet. 42, 355–360 (2010). 20208535 10.1038/ng.546 PMC2931336

[b68] Benjamini Y. & Hochberg Y. Controlling the false discovery rate–a practical and powerful approach to multiple testing. J. R. Stat. Soc. Series B Stat. Methodol. 57, 289–300 (1995).

