# Corrigendum: Open access resources for genome-wide association mapping in rice

**DOI:** 10.1038/ncomms11346

**Published:** 2016-04-20

**Authors:** Susan R. McCouch, Mark H. Wright, Chih-Wei Tung, Lyza G. Maron, Kenneth L. McNally, Melissa Fitzgerald, Namrata Singh, Genevieve DeClerck, Francisco Agosto Perez, Pavel Korniliev, Anthony J. Greenberg, Ma. Elizabeth B. Naredo, Sheila Mae Q. Mercado, Sandra E. Harrington, Yuxin Shi, Darcy A. Branchini, Paula R. Kuser-Falcão, Hei Leung, Kowaru Ebana, Masahiro Yano, Georgia Eizenga, Anna McClung, Jason Mezey

Nature Communications 7: Article number: 10532 10.1038/ncomms10532 (2016); Published: 04202016; Updated: 04202016

The affiliation details for Paula R. Kuser-Falcão are incorrect in this Article. The correct affiliation details for this author are given below:

Embrapa Agriculture Informatics, 13083-886 Campinas, Brazil.

