# Supplementary material for: Open access resources for genome-wide association mapping in rice
Source: Nat Commun. 2016 Feb 4;7:10532. doi: 10.1038/ncomms10532 (PMC4742900; doi:10.1038/ncomms10532)
Supplement: Supplementary Figures, Supplementary Tables and Supplementary References — Supplementary Figure 1-5, Supplementary Tables 1-2 and Supplementary References [file ncomms10532-s1.pdf]

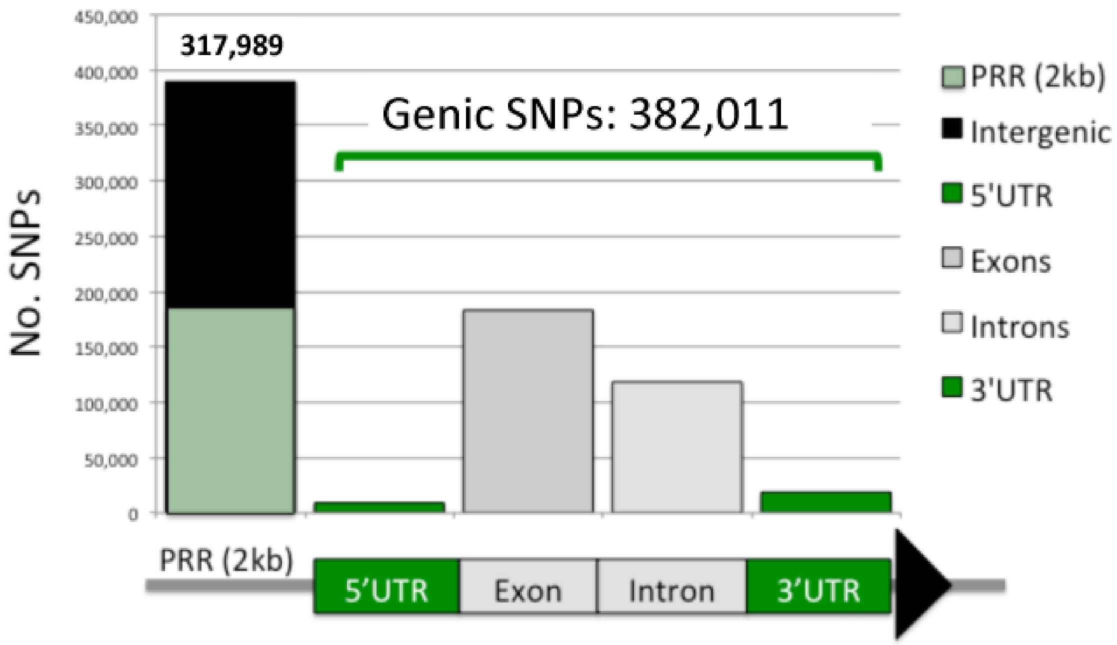

Functional domain	No. SNPs on HDRA	% SNPs on HDRA
**Total Genic**	**317,989**	**45%**
Exon	176,370	55%
Introns	114,876	36%
5'UTR	8,797	3%
3'UTR	17,946	6%
**Intergenic**	**382,011**	**55%**
PRR (2 kb)	190,616	50%

Supplementary Figure 1: Functional annotation of 700,000 SNPs on the HDRA. The bar graph and table illustrate the distribution of HDRA SNPs according to the functional annotation of the rice genome sequence (MSU v7). There are a total 115,885 non-synonymous SNPs located in 35,508 gene models, and as a whole, HDRA SNPs map within 91% of the 39,045 non-TE annotated genes in MSU v7.

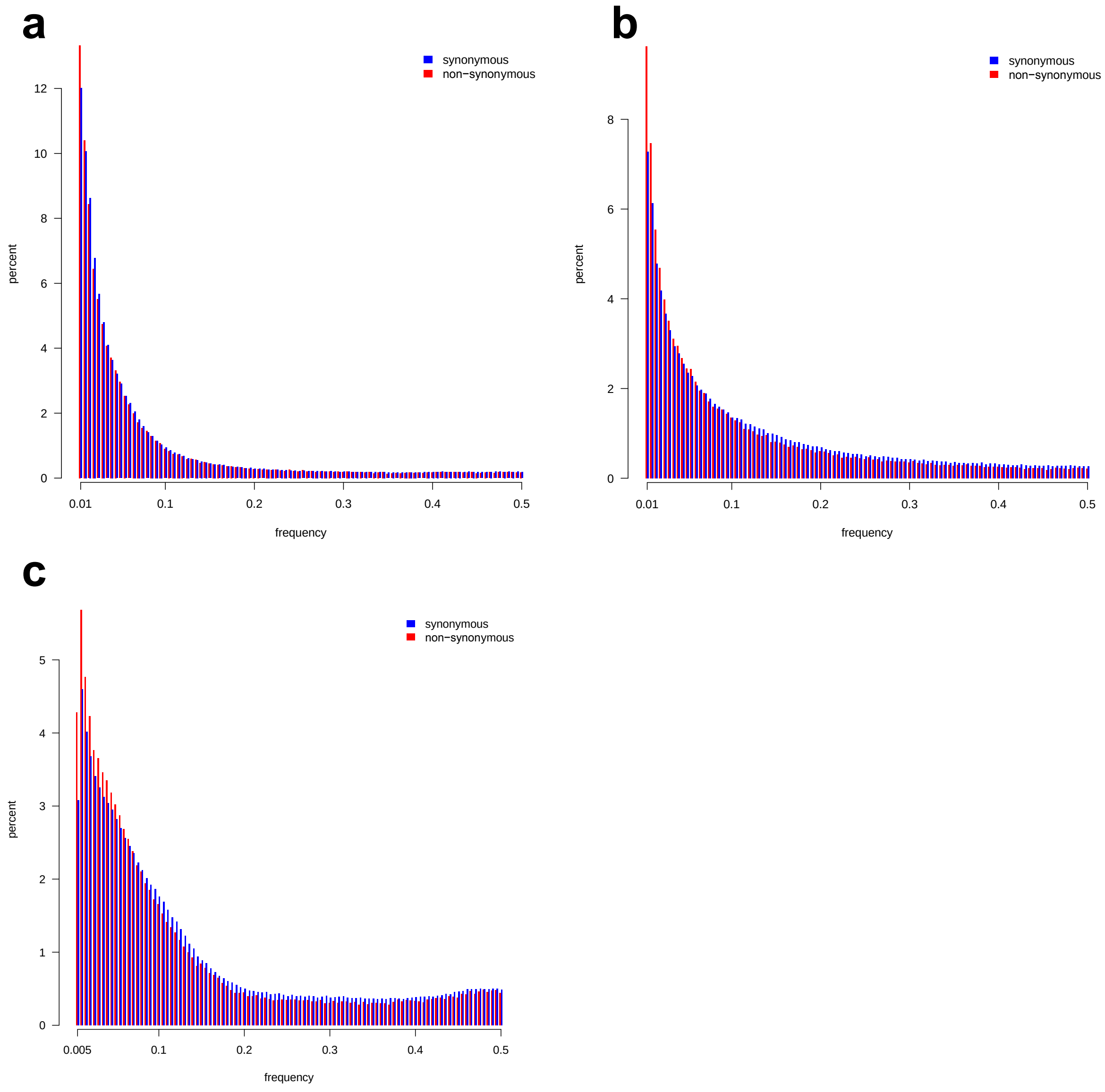

Supplementary Figure 2: Site Frequency Spectra for synonymous and non-synonymous SNPs in different subpopulation groups. In each plot, the x axis shows SNP frequency calculated across 100 bins; the y axis indicates percent of SNPs found in each bin. Synonymous SNP frequency shown as blue bars, non-synonymous SNP frequency as red bars in **(a)** the *Japonica* varietal group; **(b)** the *Indica* varietal group and **(c)** ALL subpopulations together.

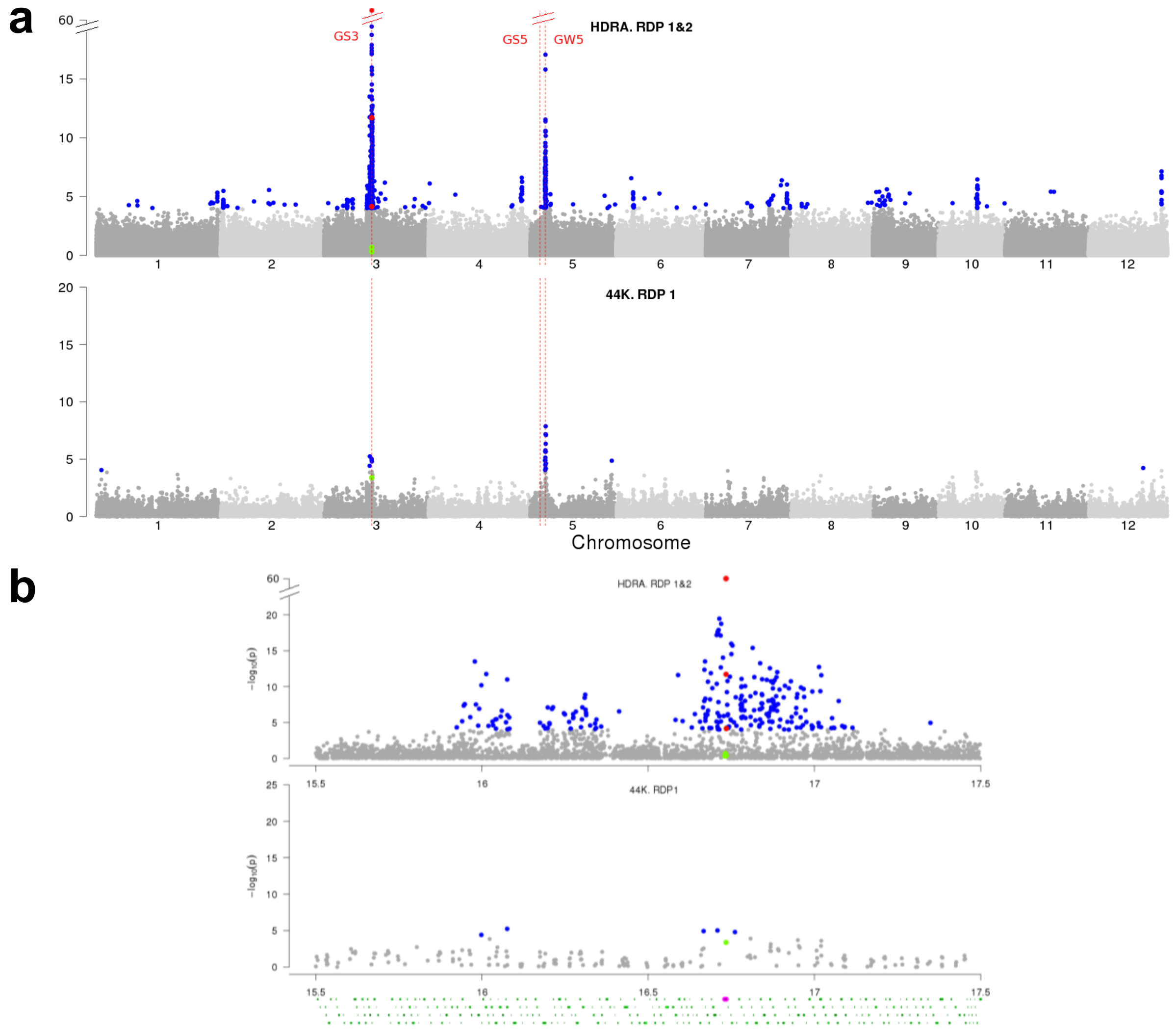

Supplementary Figure 3: GWAS for grain length comparing the new high-density rice array (HDRA) and the 44,000 SNP chip. (A) GWAS results using the HDRA on RDP1&2 (top) and the 44K on RDP1 (bottom). On each Manhattan plot, the x axis shows SNPs along each chromosome; y axis is the −log10 (*p* value) for the association. **(B)** Zoom-in on the major GWAS peak for grain length on chromosome 3 (15.5 to 17.5 Mb) using the HDRA on RDP1&2 (top) and the 44K on RDP1 (bottom). Green circles underneath the zoom-in plots represent gene models; the gene highlighted in magenta is *GS3*. In both **(A)** and **(B)**, significant SNPs are colored blue. The HDRA contains 6 SNPs within the coding region of *GS3*, while the 44K has one. These are highlighted in green if they have nonsignificant *p* values and in red if they have significant *p* values. The HDRA SNPs highlighted are [SNP name (*p* value): SNP-3.16728637 (0.50); SNP-3.16730230 (0.21); SNP-3.16732086 (1.05E-59); SNP-3.16732418 (1.96E-12); SNP-3.16733169 (7.02E-05) and SNP-3.16733346 (0.42). The 44K SNP highlighted is wd3000590 (4.20E-04).

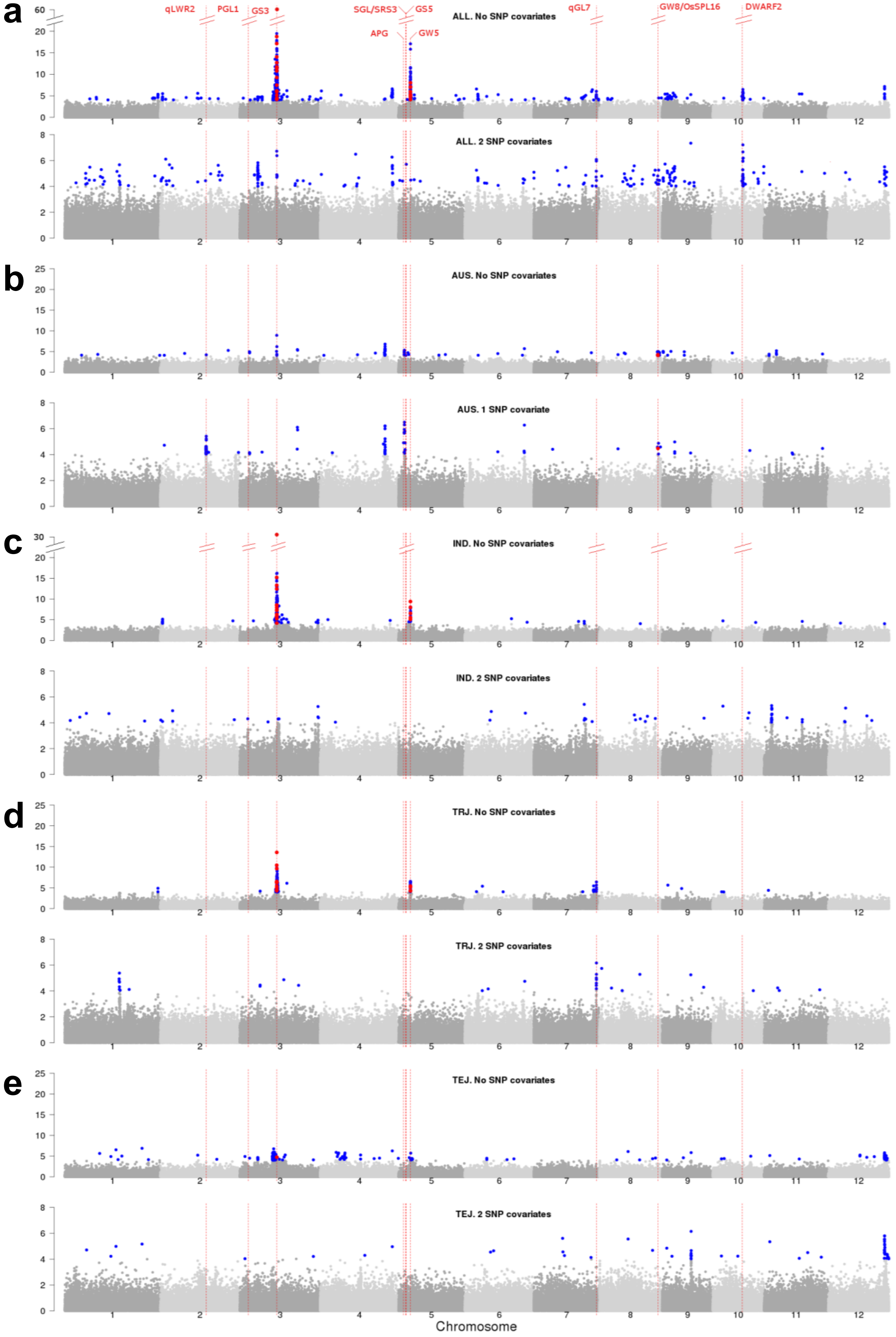
a
qLWR2
PGL1
GS3
SGL/SRS3
GS5
APG
GW5
qGL7
GW8/OsSPL16
DWARF2
ALL. No SNP covariates
ALL. 2 SNP covariates
b
AUS. No SNP covariates
AUS. 1 SNP covariate
c
IND. No SNP covariates
IND. 2 SNP covariates
d
TRJ. No SNP covariates
TRJ. 2 SNP covariates
e
TEJ. No SNP covariates
TEJ. 2 SNP covariates
Chromosome

Supplementary Figure 4: Subpopulation-specific GWAS for grain length with and without SNP covariates. Two SNP covariates were used in subpopulations that showed two major peaks on chromosomes 3 and 5. The most significant SNP for each peak (on chromosome 3 and 5) was used as covariate in the model for: *ALL*, *indica*, *tropical japonica* and *temperate japonica*. Only one SNP covariate was used for *aus*, which didn't display a peak over *GW5*. Pairs of Manhattan plots show: **(A)** RDP1&2 *ALL* with 0 SNP covariates (top) and with 2 SNP covariates: SNP-3.16732086 & SNP-5.5371749. **(B)** RDP1&2 *AUS* with 0 SNP covariates (top) and with 1 SNP covariate: SNP-3.16709886 (bottom). **(C)** RDP1&2 *IND* with 0 SNP covariates (top) and with 2 SNP covariates: SNP-3.16732086 & SNP-5.5359497 (bottom). **(D)** RDP1&2 *TRJ* with 0 SNP covariates (top) and with 2 SNP covariates: SNP-3.16732086 & SNP-5.5375144. **(E)** RDP1&2 *TEJ* with 0 SNP covariates (top) and with 2 SNP covariates: SNP-3.15327852 & SNP-5.5501218. On each Manhattan plot, the x axis shows the SNPs along each chromosome; y axis is the −log10 (*p* value) for the association. Significant SNPs (with *p* values at or above the 10% FDR threshold) are colored blue. Significant SNPs within a 24 Kb window surrounding candidate grain length genes are colored red. Red vertical lines indicate the position of *GS3*, *GS5* and *GW5*, and of grain length candidate genes or QTL located in the vicinity of subpopulation-specific GWAS peaks (see main text).

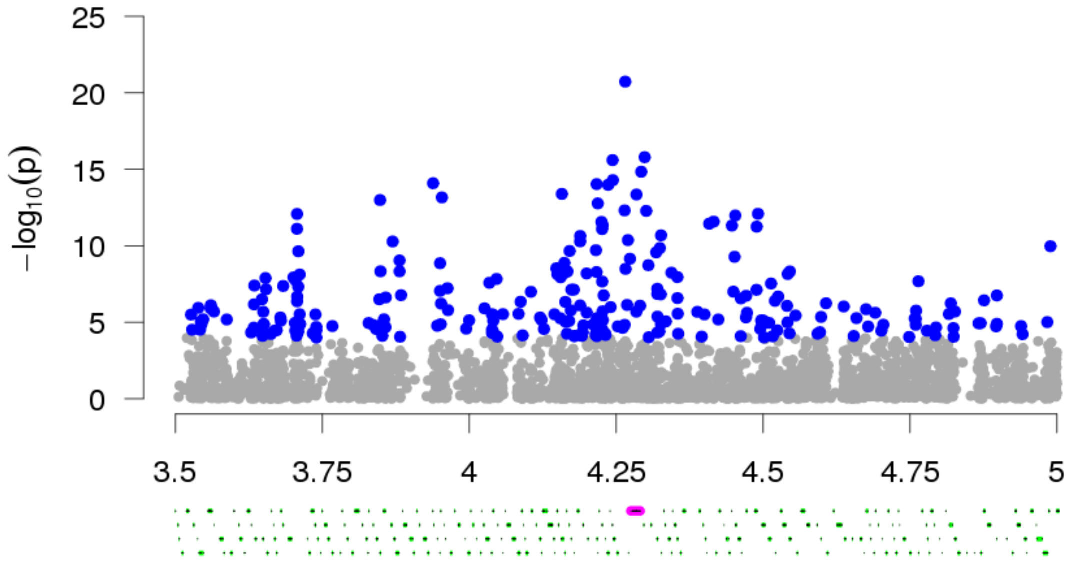

Supplementary Figure 5: Zoom-in plot showing region on chromosome 4 with significant interaction for grain length with SNP-5.5371749 at *GW5* in ALL. The x axis shows SNPs along each chromosome; y axis is the −log10 (*p* value) for the interaction. Significant SNPs (with *p* values at or above the 10% FDR threshold) are highlighted in blue. Green circles underneath the zoom-in plots represent gene models; the gene highlighted in magenta is LOC_Os04g08034, encoding a C2H2 zinc finger transcription factor. The most significant SNP in the interaction peak is SNP-4.4260961. (*p* value = 1.86E-21). LOC_Os04g08034 is located 9.5 Kb upstream of the interaction MS-SNP.

Supplementary Note 1

Development of the custom-designed High Density Rice Array (HDRA).

SNP discovery dataset. The HDRA was developed as an Affymetrix Custom GeneChip Array (www.affymetrix.com) from a SNP discovery dataset consisting of ~16M SNPs. SNPs were identified in re-sequencing data from 128 samples, including 40 wild (37 *O. rufipogon/O. nivara,* 1 *O. meridionalis,* 1 *O. officinalis,* 1 *O. punctata*) and 88 *O. sativa* (19 *temperate japonica*, 18 *tropical japonica*, 11 *aromatic/Group V*, 21 *indica*, 16 *aus*, 3 admixed) genomes. The re-sequencing data was generated using Illumina GenomeAnalyzer II, IIx, or HiSeq instruments between 2007-2010. Read length varied from 54 - 129bp paired-end reads, with coverage depths ranging from 2X-100X, with an average of 7X genome coverage in the 128 samples. SNPs were called using the PANATI alignment and genotype-calling software.

SNP selection criteria for HDRA. Selection of SNPs for the array required simultaneously choosing biologically informative SNPs while optimizing for the biochemical aspects and constraints of the Affymetrix hybridization protocol. For the latter, we required that the flanking sequence, +/- 16 bp from a candidate SNP, be invariant in the discovery panel. Also, the 33 bp sequence centered on the candidate SNP was required to have a minimum sequence complexity or randomness, lack of homopolymer runs 5 bp or longer, and GC content within 30-70%. When designing 25bp probes at different offsets from centered on the interrogated SNP, predicted melting temperature (T_m) was required to be within range 48-55°C (nearest neighbor method).

Step-wise SNP selection to tag haplotype diversity. We aimed to select the fewest number of SNPs to uniquely identify all haplotypes within a given genomic region. This was done to a first approximation via a greedy algorithm over a dynamic sliding window as follows: (1) expand the window size moving 3’ along the chromosome until all samples in the discovery panel have a unique haplotype, disregarding singleton SNPs. Additionally, the window must expand to contain at least 100 new SNPs and be at least 10 kb wide but at most 50 kb wide, (2) select the SNP that generates the highest number of unique haplotypes when combined with all SNPs already selected for the window, selecting one at random initially if needed. In the frequent case of a tie, higher minor allele frequency SNPs are chosen over lower ones, or chosen randomly if this is also a tie. (3) Repeat step (2) until all samples have a unique haplotype with the set of selected SNPs or there are no further candidates that can be selected. (4) Shrink the window from the 5’ end until the number of distinct haplotypes is at most $1/5^{th}$ the discovery panel depth. (5) Return to step (1) to define the next window. The SNPs selected in the previous window that are in the overlap with the next window are preselected for step (2) in the next window. (6) Repeat for all chromosomes. In this fashion, the SNP selection algorithm dynamically adjusts for mutation and recombination rate variation across the genome, using large windows where LD is extensive or diversity is low, and small windows where either recombination or mutation rate is high and haplotype complexity is rich. In addition to this, any non-synonymous SNPs that satisfied all technical criteria were also included.

Due to the deep divergence between subpopulations as well as high diversity within the *indica* and *aus* subpopulations, and even higher diversity of the related wild species *O. rufipogon*, it became clear that

the constraint of invariant flanking sequence 16 bp +/- of a candidate SNP would eliminate the vast majority of SNPs from inclusion in the selection pool. To address this concern, we undertook SNP selection *within each subpopulation.* This meant that SNPs segregating within each subpopulation could be selected based on their haplotype informativeness for that subpopulation (as described above) without being "blocked" by another SNP that is found segregating only in divergent subpopulations. SNP selection proceeded serially from the least diverse subpopulation *(temperate japonica*) to the most diverse *(indica*), followed by *O. rufipogon.* SNPs selected in the previous subpopulations which also segregated in subpopulations considered subsequently, were considered "preselected" by step 2 above if they satisfied the flanking sequence invariance constraint for that subpopulation. This design gave rise to considerable complexity in genotype calling, as SNPs were designed where the flanking sequence invariance constraint was known to be violated, but only for certain SNPs in certain subpopulations.

Offset probe design. For all SNPs selected for the array, we designed 6 probes as 3-A allele and 3-B allele probes at offsets from center ranging from -6 to +6. A small fraction of SNPs have only 4 probes (2-A, 2-B). For all SNPs, the "A" allele is the reference allele (Os-Nipponbare-Reference-IRGSP-1.0 assembly).

Invariant Probes. Additionally, we designed 25 bp probes complimentary to invariant regions of the genome such that every 500 kb window had at least two designed features, either interrogated SNPs or invariant probes. We use fluorescent intensity measured at invariant probes to normalize systematic differences between samples, as described below. The invariant probes are also intended to facilitate copy number analyses in future studies.

Internal Replicates (RSNPs). Of 1,021,725 SNP assays designed on the array, 998,069 are unique. The remaining 23,656 SNP assays are internal replicates at random, physically distinct locations on the array for the purposes of sample QC and error rate estimation. Concordance of a SNP with its internal replicate RSNP is an indication of repeatability of the assay and integrity of the overall physical outcome of the combined steps of DNA isolation, molecular sample preparation, labeling, array hybridization and washing, and image scanning. The average concordance across all RSNPs provides a direct estimate of the genotype error rate for each sample automatically without need for any full technical replicates to estimate this for the entire dataset as a single number. The standard practice of performing full technical replicates to produce an averaged estimate of the reliability of the total dataset provides no individual sample error rate or reliability estimate with which to identify and exclude individual failed samples, except for those replicated. From a small number of full technical replicates, this sample-wide error rate estimate was found to be sufficiently accurate. This proved critical in further identifying and excluding failed samples.

Target labeled-probes preparation and hybridization protocols. A total of 50 ng of high-quality rice genomic DNA was used as template for labeled-probe generation. Total genomic DNA was amplified using the Qiagen REPLI-g Midi kit; the resulting 40 ug of whole genome amplification (WGA) product was fragmented with 0.25 U of DNaseI at 37ºC for 35 min, 3ul of the digested product was checked on 4% agarose gel to confirm the fragment size at ~50 to 150 bp. Probe labeling followed the manufacturer's recommendation for the Affymetrix SNP assay kit. The hybridization protocol was the same as described in Zhao et al (2011).

Genotype calling. During the genotype calling procedure, raw probe intensities are extracted from Affymetrix .CEL files, converted to log scale. For all SNPs that had sufficient minor allele frequency in the discovery panel (at least 5 minor homozygous alleles), the intensity distribution of individual probes was analyzed to determine which probes had the ability to discriminate between A and B alleles. Probes that did not have at least 70% statistical power for allele discrimination were masked out. We refer to this as *probe level masking*. Some SNPs fail at this stage as a result of either all of the A allele probes or all of the B allele probes for that SNP being masked out (see SNP QC below). Subsequent statistics are based on genotype calling success, disregarding the masked out probes. In following steps, any masked probe is not included in the averaged A and B log intensity levels.

Next, Affymetrix Power Tools (APT) software (Affymetrix, Inc.) was used to extract individual probe intensities from Affymetrix .CEL files for each sample. First, probe intensities at invariant probes were extracted for all samples and converted to log scale. A simple pre-genotype-calling normalization routine was employed where a "reference" invariant intensity for each probe was derived as the average across all samples for the probe. Then, for each sample, we calculated factors m and b for the linear adjustment $\boldsymbol{y} = m\boldsymbol{x} + b$ such that if $\boldsymbol{x}$ is the vector of invariant intensities observed for a sample, the adjusted or normalized invariant intensities $\boldsymbol{y}$ will have the same mean and standard deviation as the distribution of reference invariant intensities, using a standard linear regression model. Only the factors m and b for each sample are relevant from this step. At this stage we also considered the squared correlation coefficient between the reference invariant intensities and the sample's invariant intensities. Any sample with $r^2 < 0.75$ was considered a failed reaction and discarded from further processing. Manual review of array scan images for samples failing at this stage revealed either very low signal level or the presence of large, bright artifacts or dark spots where either noise level was at saturation of the dynamic range, or signal level not different from the noise floor, covering a significant portion of the array. This step was then repeated with the failed samples from the previous iteration removed from determining the reference intensities and the m and b parameters re-estimated. The individual probe intensities for each SNP were then extracted, converted to log scale, adjusted by m and b for each sample from the second iteration, and output to a file which could be loaded by the genotype calling program ALCHEMY (Wright et al., 2010).

Genotype calling was then performed by ALCHEMY v1.07 as described in Wright et al. (2010). ALCHEMY's internal bimodal normalization method was used in addition to the external normalization step described above. Expectation-Maximization based automatic estimation of inbreeding coefficients was disabled and inbreeding coefficients were set manually in the ALCHEMY sample map file to 0.9 for *O. sativa* samples that had been extensively purified (3 or more generations) through single seed descent, 0.8 for *O. sativa* samples that had been purified through 1 or 2 single seed descent generations, 0.75 for purified *O. rufipogon* samples, 0.7 if purification level was not known, and 0.0 or -0.2 for cross subpopulation F1 controls. ALCHEMY command options set the bivariate t-distribution degrees of freedom to 11 (-t 11), and EM search parameters –em-start-points=100 –em-max-iterations=200. Note that –em-start-points=100 in v1.07 directs ALCHEMY to stop searching for a better EM solution after 100 successive random start points have failed to produce a better solution, not to try exactly or at most 100 start points. Higher settings did not produce significantly better results. ALCHEMY will produce genotype calls at all samples at all SNPs, each call with an associated posterior probability of correctness (or 1 – probability of error). This posterior probability of the simple empirical Bayes model for genotype

calling that follows EM estimation of its parameters has been previously shown to be accurate assuming the model fits the data. In the final dataset, any genotype call with posterior probability < 0.70 was considered "no call" (treated as missing data in analyses), and reported call rates refer to the proportion of snps with genotype calls at or above the 0.70 threshold, averaged across all samples, except where noted below.

Sample Quality Control and Inclusion Criteria. For the purposes of assessing minimum assay quality criteria for inclusion of a sample in the final dataset, we focused first on a set of "core SNPs" on the array that do *not* have polymorphisms in the flanking sequence for either allele, in any subpopulation, that would interfere with probe hybridization and confound sample QC with sample subpopulation. A core SNP must satisfy two criteria: (1) the minor homozygote genotype has been observed the discovery panel in at least two lines, and (2) there were no other SNP calls, where the minor homozygote is also observed at least twice, within 16 bp 5' or 3' from the interrogated SNP position. These SNPs are less likely to have higher no-call rates and/or error rates correlated with sample subpopulation and are therefore most suitable for assessing overall quality of each sample's assay outcome as well as for determining or confirming the subpopulation of each sample. Not including RSNPs (see below), 318,653 SNPs satisfy these criteria. Of these, 265,449 were analyzed for genotype calling with ALCHEMY after probe level filtering was applied. Although these may be considered *a priori* the most robust set of SNPs designed on the array, the criteria outlined above do not in any way consider biochemical or molecular interaction aspects of these assays that may be predictive of higher chances of assay conversion. Thus, we define a "confirmed core" based on the empirical results from actual array assay outcomes, discarding core SNPs that have either a call rate less than 60%, or a minor allele count less than 10. This eliminated 12,826 core SNPs. We then ranked the remaining SNPs by their expected error rate calculated as the sum of posterior genotype call error estimates produced by ALCHEMY divided by the number of samples, and retained the top 200,000 SNPs as the confirmed core.

To identify and exclude samples with poor assay outcome, we estimated each sample's genotype error rate using the set of internally replicated SNPs or RSNPs which satisfied the confirmed core criteria. For each sample, we compared the genotypes at each SNP/RSNP pair with ALCHEMY posterior call probability > 0.5 at both. For a sample with ***n*** such comparisons and ***m*** discordances, we crudely estimated the sample's error rate as $(m + (n\text{-}m)*(m/2n)^2/2)/2n$. The second term in the numerator is a correction for the case where errors at both replicates are concordant by chance. It is otherwise presumed that for a discordant pair, only one is erroneous. Assays with an RSNP estimated error rate of 12.7% or higher were excluded from the final dataset. The inclusion threshold was chosen at the point of inflection where the estimated sample error rate began to sharply increase when the samples were ordered by increasing sample error rate. Of 2,089 assays that passed external normalization steps and were called by ALCHEMY, 254 were excluded because of poor assay outcome as determined by this procedure. The resulting set of 1,835 assays represents the QC-passed sample set but not the final dataset, as this set still contains technical replicates, biological replicates, control samples and F1 for Mendelian trios, as well as other samples not intended for use in GWAS analyses.

SNP Quality Control and Conversion. After determining the QC-passed sample set based on the confirmed core SNPs, we identified the larger set of "converted" or QC-passed SNPs on the array by a very similar approach to how the confirmed core was selected from the core SNPs. As mentioned

previously, of 1,021,725 SNP assays designed on the array, 998,069 are unique. After screening to mask out probes that failed to demonstrate allele specific discrimination, 828,626 SNP assays had at least one viable probe per allele and thus could be analyzed by ALCHEMY. 808,008 SNPs were unique. We then applied the same minimum segregation and call rate requirements as the confirmed core set of at least 10 minor alleles (this could be 5 minor homozygotes, or 10 heterozygotes) and a call rate of 60% or higher. Of the unique SNPs, 756,251 satisfy these minimums. (NOTE: 70% minimum call rate: 748,968; 75% minimum call rate: 734,481; 80% minimum call rate: 683,119). We then ranked these SNPs by their expected error rate based on the ALCHEMY posterior call probabilities, as described for the confirmed core SNP set. The top 700,000 unique SNPs were retained as the final QC-passed SNP set. The expected error rate for each SNP was calculated over QC-passed samples only as described above. By performing sample QC using a smaller, more robust set of SNPs uncomplicated by subpopulation divergence, and SNP QC over QC-passed samples only, the mutually confounding effects of failed SNPs on sample QC and failed samples on SNP QC is at least partially mitigated.

Supplementary Note 2

Resources

Seed Availability. Seeds from the RDP1 and RDP2 are available as homozygous genetic stocks from (1) the International Rice Genebank at IRRI, Philippines (irri.org/our-work/seeds), (2) for researchers in the US, seeds may be requested from the USDA-ARS Genetic Stocks-*Oryza* (GSOR) Stock Center (www.ars-grin.gov/npgs). Seeds from the NIAS diversity panel are available upon request at (http://www.gene.affrc.go.jp). For a detailed list of accessions, including genebank accession IDs, variety/common name, country of origin, and subpopulation identity based on FastSTRUCTURE[1] analysis, see Supplementary Data 1.

HDRA Data Files. The complete set of data files related to this publication is available for download at ricediversity.org/data. This includes the phenotypic data on grain length in the RDP1&2, the 700,000 HDRA SNPs discovered, which are also available from NCBI dbSNP DB (batch ID 1062024) and GEO (Accession ID: GSE71553), PLINK formatted genotype files, the associated germplasm sample map file, and GWAS results files related to analyses discussed herein.

GWAS Pipeline. The software pipeline developed for GWAS analysis consists of Python (v2.6-2.7) and R (v2.15) scripts that automate several pre- and post-processing steps required for linear mixed model analysis by EMMAX[2] (version 'emmax-beta-07Mar2010'). Given PLINK[3] (v1.9; http://pngu.mgh.harvard.edu/purcell/plink/) formatted genotype and phenotype files, this pipeline will

match phenotype to genotype samples, filter by MAF and/or MAC, filter by missing data parameters, create PC covariate files, and create the kinship matrix required for analysis. After EMMAX completes, the pipeline uses R[4] to generate Manhattan and Quantile-Quantile plots for each of the traits submitted and outputs an allele effect summary file for most significant SNPs. The GWAS Pipeline is run from the command line in a Linux/UNIX environment. The pipeline code is available as Supplementary Software and can also be downloaded from ricediversity.org/tools.

GWAS Viewer. The GWAS Viewer is a web application written in PHP, JavaScript, the HighCharts charting library (highcharts.com) with a supporting MySQL database (Figure 7; www.mysql.com). This graphical data browser allows researchers to view –log *p* value data within a genomic context and displays Os-Nipponbare-Reference-IRGSP-1.0/MSU7 gene annotation features that underlie potential regions of interest. Each plotted point can reveal more information about the data value and related SNP (by clicking or hovering the mouse over the dot) and includes a hyperlink to the Rice Diversity UCSC Genome Browser (ricediversity.org) for closer examination of a SNP of interest within the genome annotation. The GWAS Viewer allows up to six plots to be loaded on-screen for any analysis that has been pre-loaded into the database backend, and plots can be zoomed synchronously for dynamic comparisons. The user can export and print any plots rendered in a session. To access the GWAS Viewer, please visit ricediversity.org/tools.

Allele Finder. The Allele Finder was developed as part of the Rice Diversity project as a means to query and browse genotype data in a user-friendly graphical interface (Fig. 7). The Allele Finder is a web application implemented primarily in Python and Javascript with an SQL backend. Genotypes are retrievable by genomic location for different sets of germplasm based on sample name or subpopulation. Alleles are displayed color-coded by nucleotide and can be masked out according to different attributes (e.g. mask/show heterozygous genotypes). Data are displayed to users in tabular form with genotypes encoded as A/G/C/T or A/B and can be exported or downloaded in plain-text format. The Allele Finder also displays MSUv7 gene models that are hyperlinked to our UCSC Genome Browser so the user can easily click for genomic context information. To access the Allele Finder, please visit ricediversity.org/tools.

UCSC Genome Browser. We deployed a local mirror of the UCSC Genome Browser software[5] (genome.ucsc.edu) for analysis of the HDRA SNP data in a genomic context (Fig. 7). Both the Os-Nipponbare-Reference-IRGSP-1.0/MSU7 and the RAP-DB (rapdb.dna.affrc.go.jp) genome reference assemblies have been loaded. We created tracks for the HDRA SNP dataset, as well as for the Rice

Diversity 44K SNP and 1536 SNP sets, and 384 OPA breeder's mini-chips[6]. We have adapted other browser format tracks to display SNP annotations such as putative amino acid changes and other functional annotations. In addition to the default tracks, users can create and load their own annotation tracks for displaying custom annotations and or datasets that map to the IRGSP-1.0 coordinates. To access the local mirror of the UCSC Genome Browser loaded with rice diversity data, please visit ricediversity.org/tools.

Supplementary References

1 Raj, A., Stephens, M. & Pritchard, J. K. fastSTRUCTURE: Variational Inference of Population Structure in Large SNP Data Sets. *Genetics* **197**, 573-589 (2014).

2 Zhang, Z. *et al.* Mixed linear model approach adapted for genome-wide association studies. *Nat Genet* **42**, 355-360, doi:10.1038/ng.546 (2010).

3 Purcell, S. *et al.* PLINK: a tool set for whole-genome association and population-based linkage analyses. *American journal of human genetics* **81**, 559-575, doi:10.1086/519795 (2007).

4 The R Core Team. *R: A Language and Environment for Statistical Computing*, http://www.R-project.org (2014).

5 Kent, W. J. *et al.* The human genome browser at UCSC. *Genome Res* **12**, 996-1006, doi:10.1101/gr.229102 (2002).

6 Thomson, M. *et al.* High-throughput single nucleotide polymorphism genotyping for breeding applications in rice using the BeadXpress platform. *Molecular Breeding* **29**, 875-886, doi:10.1007/s11032-011-9663-x (2012).
